# Screening and Identification of BMP5 as a Key Regulatory Gene for hPSCs Transcardiomyocyte Differentiation

**DOI:** 10.1155/sci/5540587

**Published:** 2026-04-24

**Authors:** Hong Zhang, Simei Tu, Chang Xu, Ruoshi Wu, Jiayi Wu, Xin Huang, Xiaoqin Luo, Xiaowei Luo, Xinjian Qu

**Affiliations:** ^1^ Institute of Marine Drugs/Guangxi Key Laboratory of Marine Drugs, Guangxi University of Chinese Medicine, Nanning, 530200, China, gxtcm.com; ^2^ Guangxi University Engineering Research Center of High-efficient Utilization of Marine Traditional Chinese Medicine Resources, Guangxi University of Chinese Medicine, Nanning, 530200, China, gxtcm.com; ^3^ School of Life and Pharmaceutical Sciences, Dalian University of Technology, Dalian, Liaoning, China, dlut.edu.cn; ^4^ Nanning Hospital of Foresea Life Insurance, Nanning, 530200, Guangxi, China

**Keywords:** BMP5, cardiomyocytes, hPSC differentiation, human pluripotent stem cell

## Abstract

**Background:**

Human pluripotent stem cells (hPSCs)‐derived cardiomyocytes have tremendous advantages in generating cell models that are suitable for the study of cardiovascular diseases and their treatment. However, there is a lack of systematic screening and analysis of important genes for inducing hPSCs differentiation into cardiomyocytes.

**Methods:**

Gene Expression Omnibus (GEO) databases were used to explore the key genes involved in hPSCs differentiation into cardiomyocytes. Then, the induction of human embryonic stem cells (hESCs) differentiation into cardiomyocytes was verified by alkaline phosphatase (ALP) detection, real‐time PCR, western blotting and immunofluorescence experiments. Furthermore, BMP5 overexpression and CRISPER/Cas9‐mediated knockout of BMP5 were performed to determine the important role of BMP5 in the differentiation of hESCs into cardiomyocytes.

**Results:**

Through differential expression analysis of high‐throughput sequencing datasets from four stages of hPSCs differentiation into cardiomyocytes (undifferentiated hPSCs, mesoderm cells, early cardiomyocytes, and cardiomyocytes), 10 key differentially expressed genes (DEGs) were ultimately identified. Weighted gene co‐expression network analysis (WGCNA) and protein–protein interaction (PPI) network analysis subsequently revealed that BMP5 interacted with the transcriptional regulator NKX2‐5. During the induction of hESCs differentiation into cardiomyocytes, the expression of BMP5 was significantly increased in the mesoderm, early cardiomyocyte, and cardiomyocyte stages compared with that in the hESCs stage. After overexpression of BMP5 in hESCs and induction of cell differentiation into cardiomyocytes, the number of myocardial beats and the expression levels of cardiomyocyte marker proteins were significantly greater than those in nontransfected hESCs‐derived cardiomyocytes. CRISPR/Cas9‐mediated knockout of BMP5 in ESCs and then cells were induced to differentiate towards cardiomyocytes, transcriptome sequencing results revealed a significant reduction in the expression levels of genes related to myocardial development.

**Conclusion:**

The role of BMP5 in the differentiation of hESCs into cardiomyocytes was explored, and findings demonstrated that BMP5 promoted the differentiation of hESCs into cardiomyocytes.

## 1. Introduction

Myocardial disease is a serious disease that threatens the life and health of humans worldwide [1]. An important factor in current strategies for the treatment of myocardial disease is the acquisition of cardiomyocytes with normal physiological function [2]. It has been reported that human pluripotent stem cells (hPSCs)‐derived cardiomyocytes play important roles in the fields of cell therapy, disease modeling, and drug development for treating myocardial disease [3]. The differentiation of hPSCs into cardiomyocytes is regulated by multiple genes and other factors. A prerequisite for obtaining large amounts of hPSCs‐derived mature cardiomyocytes is a deep understanding of the molecular mechanisms of hPSCs differentiation into cardiomyocytes [4]. It has been reported that the Wnt signaling pathway, bone morphogenetic proteins (BMPs), and the activin/nodel signaling pathway play key roles in inducing the differentiation of hPSCs into mesoderm cells [5–7].

With the rapid advancement of transcriptomics and bioinformatics, researchers can now comprehensively analyze changes in gene expression levels during specific developmental stages and identify key genes associated with physiological functions. One powerful tool for this purpose is weighted gene co‐expression network analysis (WGCNA), a systems biology algorithm designed to construct gene co‐expression networks. WGCNA excels at processing complex, large‐scale high‐throughput sequencing data while uncovering relationships between genes across different samples. This approach is based on the principle that genes with similar functions often exhibit comparable expression patterns [8]. Due to its effectiveness in pinpointing hub genes—such as biomarkers capable of distinguishing stem cells or tumor cells—WGCNA has gained widespread adoption in the biomedical field internationally [9, 10].

To date, there has been no systematic study of the classification, expression levels, and pivotal role of genes at specific stages during the differentiation of hPSCs into cardiomyocytes. Therefore, in this study, bioinformatics methods were used to analyze the transcriptome data during hPSCs differentiation into cardiomyocytes, screen key genes involved in the process of hPSC differentiation into cardiomyocytes, and experimentally verify the roles of key genes in the differentiation process to provide a research basis for obtaining mature cardiomyocytes.

## 2. Materials and Methods

### 2.1. Acquisition of Gene Expression Data for Induced Pluripotent Stem Cell (iPSC) Differentiation Into Cardiomyocytes

Gene expression profile data (GSE137920) pertaining to iPSC differentiation into cardiomyocytes were retrieved from the Gene Expression Omnibus (GEO) database (https://www.ncbi.nlm.nih.gov/geo/). The dataset was meticulously curated to encompass data from four distinct stages of iPSC differentiation into cardiomyocytes [11], which included three samples each from undifferentiated iPSCs (D0), mesodermal cells (D2), early cardiomyocytes (D7), and fully differentiated cardiomyocytes (D14). These datasets were subsequently organized into gene expression matrices for further analysis.

### 2.2. Pathway Enrichment Analysis of Important Modules

Gene Ontology (GO) and Kyoto Encyclopedia of Genes and Genomes (KEGG) analyses were performed on the core genes to investigate their primary functions and involvement in signaling pathways. The R software package org.Hs.eg.db Version 3.12.0 was utilized to convert gene names into gene IDs, followed by enrichment analyses of GO functions and KEGG signaling pathways for differentially expressed genes (DEGs) between D0 and the subsequent stages, D2, D7, and D14, using the clusterProfiler v3.0.4 package. The results were then visualized using ggplot2 Version 3.3.2 and enrichplot Version 1.10.1. Furthermore, the Reactome FIPlugIn within the Cytoscape 3.7.2 platform was employed to analyze the pathways associated with genes in significant modules, with analysis criteria established at a false discovery rate (FDR) ≤0.05 and *p*  ≤ 0.01 [11].

### 2.3. Construction of a WGCNA System for the Differentiation of iPSCs Into Cardiomyocytes

WGCNA identifies genes exhibiting similar expression patterns based on differential gene expression profiles and organizes them into modules. The characteristics of these modules and the hub genes within them were determined, with each module being correlated to other modules or external sample features to identify core genes serving as biological markers. The gene expression matrix from the four stages of iPSC differentiation into cardiomyocytes (D0, D2, D7, and D14) was processed in R software, designating genes with average expression levels exceeding the overall mean as highly expressed genes, which were then subjected to analysis using the WGCNA (1.69) plugin. Initially, sample clustering analysis and soft thresholding were conducted. The adjacency matrix was transformed into a topological overlap matrix, and genes were clustered into distinct modules via dynamic tree cutting, with a minimum module size parameter set at 30. Modules that exhibited proximity were merged into a new module, applying a cutoff of 0.25, while modules with a similarity greater than 0.75 were combined. Ultimately, the significant gene module was selected, and the Cytohubba plugin in Cytoscape 3.7.2 was utilized to visually analyze the interacting genes within the module and to identify the hub genes.

### 2.4. Venn Diagram Analysis of DEGs and Important Modules

The DEGs in the D2, D7, and D14 sample datasets were identified through the limma package in the R software. Genes meeting the cutoff criteria of |log_2_FC| ≥ 2.0 and FDR < 0.05 were regarded as DEGs. FDR represents the adjusted *p*‐value and log_2_FC represents the ratio of expression levels between experimental and control group samples. The overlapping genes between the DEGs and the co‐expressed genes in the functional modules obtained via WGCNA of the D2, D7, and D14 sample data were considered the hub genes. In this study, hub gene acquisition was performed by the VennDiagram package in the R software to construct a Venn diagram [11].

### 2.5. Construction of the Protein–Protein Interaction (PPI) Network of Differentially Co‐Expressed Genes

The differentially co‐expressed genes were searched for by the STRING database (http://string-db.org/) to identify the interaction relationships between the proteins encoded by the differentially co‐expressed genes and to construct a network with a standard interaction score ≥0.9. The obtained PPI network was subsequently visualized by the CytoHubba plug‐in in the Cytoscape software 3.7.2, and the top 10 genes in the network were identified as hub genes by the maximal clique centrality (MCC) algorithm [11].

### 2.6. Cell Culture

hiPSCs and human embryonic stem cells (hESCs) were purchased from Beijing Cellapy Biotechnology Company. PGM1 PSC medium, PSCeasy recovery medium, PSCeasy base working solution, and PSCeasy digestion solution were purchased from Beijing Cellapy Biotechnology Company.

A cell suspension was pipetted into a sterile 15‐mL centrifuge tube, 4 mL of PSCeasy recovery medium was added, and the mixture was centrifuged at 200 × g for 5 min. Then, the supernatant was removed, 1 mL of PSCeasy recovery medium was slowly added, and the mixture was mixed well. The cell suspension was inoculated into a culture flask, and after 1 day of incubation, the culture was continued with a fresh PGM1 PSC medium, followed by the addition of fresh PGM1 PSC medium every day.

### 2.7. pAd‐BMP5‐EGFP Construct and Transfection

To construct recombinant adenovirus vector carrying human BMP5 gene and transfect hESCs. Briefly, pBMP5‐EGFP and pAdTrack‐CMV plasmid were digested using endonuclease Kpn Ⅰ and Xho ⅠI. Then, the digest fragment of BMP5‐EGFP containing the sites of restriction endonuclease Kpn Ⅰ and Xho ⅠI was inserted into shuttle plasmid pAdTrack‐CMV. After analysis of restriction endonuclease and confirmation by sequencing, the recombinant shuttle plasmid pAdTrack‐CMV‐BMP5‐EGFP was linearized by PmeⅠ and then transformed into *E. coli*. BJ5183, which was transformed by adenoviral backbone plasmid pAdEasy‐1. The recombinant plasmid pAd‐BMP5‐EGFP obtained from screening was confirmed by PCR and restriction endonuclease analysis. The pAd‐BMP5‐EGFP plasmid was linearized by Pac Ⅰ and transfected into HEK293T via liposome. The recombinant adenovirus was packaged and amplified in HEK293T cells following three amplifications. The prepared, highly expressed Ad‐BMP5‐EGFP was transfected into hESCs.

Inoculate ESCs in 6‐well culture plates at 5 × 10^4^ per well. When the cells were grown to 70% confluency, the fresh culture medium was replaced, and the virus solution was added according to the multiplicity of infection (MOI) of 0, 10, 25, 50, 100, and 200 after 2 h, and the expression of green fluorescent protein (GFP) was observed under fluorescence microscopy after 24 h.

### 2.8. CRISPR/Cas9‐Mediated Knockout of BMP5

The CRISPR/Cas9 genomic editing system was used to create BMP5 knockout (BMP5‐KO) ESCs. The stable lentivirus vectors were produced and packaged by Cyagen Company (Suzhou, China). The BMP5 gene (NCBI Reference Sequence: NM_021073; Ensembl: ENSG00000112175) is located on human chromosome 6. Seven exons are identified, with the ATG start codon in exon 1 and the TAA stop codon in exon 7 (Transcript BMP5‐201: ENST00000370830). To create a human BMP5‐KO model by CRISPR/Cas‐mediated lentivirus vector genome engineering. Exon 1 was selected as target site. The sequences are presented in Table 1. The gRNAs target sites for BMP5 deletion were as follows: LV‐U6 >(hBMP5‐gRNA‐A1)‐U6 >(hBMP5‐gRNA‐A2)‐PGK >EGFP/T2A/Puro. hESCs transduced with lentivirus and puromycin (AMRESCO, HY‐B1743S) selection (5 µg/mL) were applied after 3 days, where BMP5‐KO cells were further isolated for monoclonal cells by limiting dilution and then continued to be amplified.

**Table 1:** List of gRNA target sequences.

Names	Sequences
BMP5 KO#A1	5′‐ACTACGGAACCACGAAAGAC‐GGG‐3′
BMP5 KO#A2	5′‐CGAGATAACTGTATGCGACG‐AGG‐3′

### 2.9. Cardiomyocyte Differentiation

CardioEasy human cardiomyocyte differentiation media I, II, III and CardioEasy human cardiomyocyte differentiation basic medium were purchased from Beijing Cellapy Biotechnology Company.

A 24‐well plate was coated with the PSCeasy base working solution, and the cells were seeded and cultured in PGM1 PSC medium. The medium was changed daily until the cell confluence reached 90%. The cells were then induced to differentiate as follows. First, the PGM1 PSC medium was aspirated, and the cells were washed 2 times with 1 mL of a PBS solution. About 1 mL of cardioEasy human cardiomyocyte differentiation medium I was added to each well, and the plate was incubated for 2 days. Then, the medium was aspirated, the cells were washed twice with 1 mL of PBS, 1 mL of CardioEasy human cardiomyocyte differentiation medium II was added to each well, and the cells were cultured for 2 days. The medium was aspirated again, the cells were washed twice with 1 mL of PBS solution, CardioEasy human cardiomyocyte differentiation medium III was added to each well and then replaced every 2 days until beating cardiomyocytes appeared. The induced differentiation of the cells was observed under a microscope every day.

### 2.10. Alkaline Phosphatase (ALP) Assay

An ALP assay kit (Cat. No. P0321S) was purchased from Beyotime Biotechnology. Cells were seeded on 6‐well plates at an initial density of 3 × 10^4^ cells/well and cultured to 80% confluence. The cells were fixed with 4% paraformaldehyde for 30 min at room temperature. An ALP assay kit was used to stain cells for 30 min at room temperature on Day 14 after transfection. In total, three randomized observation views were selected, and the numbers of mineralized nodules were counted under a microscope (Zeiss Axio Observer.Z1; Carl Zeiss AG).

### 2.11. Real‐Time PCR

Total RNA was isolated from cells by using the RNAiso reagent (TaKaRa, Dalian, China). Total RNA (3 μg) was reverse transcribed with oligo(dT) primers and a reverse transcription system (TaKaRa). The primers used are shown in Table 2. The PCR products were analyzed by 1% agarose gel electrophoresis. Relative mRNA levels were determined on an ABI Prism 7500 sequence detection system with SYBR premix Ex Taq (TaKaRa). The expression of target genes was determined by the 2^−△△CT^ method, with GAPDH used as a reference gene [11].

**Table 2:** The primer sequences used for qPCR.

Gene	Primer	Sequence (5’–3’ direction)
Sox2	Forward	5’‐CAGCATGTCCTACTCGCAGCAG‐3’
	Reverse	5’‐CTGGAGTGGGAGGAAGAGGTAACC‐3’
c‐Myc	Forward	5’‐ CGACGAGACCTTCATCAAAAAC‐3’
	Reverse	5’‐CTTCTCTGAGACGAGCTTGG‐3’
cTnT	Forward	5’‐TTCTCTCAAAGACAGGATCGAG‐3’
	Reverse	5’‐GTTGGACAAAGCCTTCTTCTTC‐3’
NKX2‐5	Forward	5’‐AGAACCGGCGCTACAAGT‐3’
	Reverse	5’‐GGGTAGGCGTTATAACCGTAG‐3’
MESP1	Forward	5’‐GGAGACAGTCGTGAAGAGACATTATGG‐3’
	Reverse	5’‐ACTAGGTGGCTCTGGCAGGTTC‐3’
ISL1	Forward	5’‐ATGTGCGGAGTGTAATCAGTAT‐3’
	Reverse	5’‐ATTTGATCCCGTACAACCTGAT‐3’
BMP5	Forward	5’‐GTATCCTCGTCGCATACAGTTA‐3’
	Reverse	5’‐ATTCTTTGTAATGCCTTCGCTG‐3’
SMYD1	Forward	5’‐TGGAGTTACAAGTGGCAGGTTACAAG‐3’
	Reverse	5’‐ATTCTTTGTAATGCCTTCGCTG‐3’

### 2.12. Immunofluorescence Detection and Western Blotting

Protein expression was analyzed with a troponin T (cardiac) rabbit lgG (Proteintech), an α‐actinin rabbit mAb (Cell Signaling Technology), a rabbit anti‐GAPDH antibody (Sangon Biotech) and a goat anti‐rabbit lgG antibody (Sigma, Saint Louis, MO, USA). Immunofluorescence detection and western blot assays were conducted as previously described.

### 2.13. Graphing and Statistical Analysis

The statistical analyses were conducted utilizing Origin‐9 and GraphPad Prism 8 software. The data are presented as means ± standard deviation (S.D.). To determine the significance of intergroup differences, one‐way analysis of variance (ANOVA) followed by Tukey’s multiple comparison test was employed. The notation for significance is as follows: ns indicates no significance,  ^∗^ *p*  < 0.05,  ^∗∗^ *p*  < 0.01, and  ^∗∗∗^ *p*  < 0.001.

## 3. Results

### 3.1. Screening and Analysis of DEGs During the Differentiation of hiPSCs Into Cardiomyocytes

Gene expression datasets (GSE137920) pertaining to the differentiation of iPSCs into cardiomyocytes were retrieved from the GEO database. Data were acquired from four distinct developmental stages: the undifferentiated iPSC stage (D0), mesodermal stage (D2), early cardiomyocyte stage (D7), and cardiomyocyte stage (D14).

Using FDR <0.05 and |log_2_FC| ≥2 as the criteria for screening differential genes. A total of 2723 DEGs (comprising 959 upregulated and 1764 downregulated genes) were identified between the differentiated D2 and D0 samples. The analysis further revealed 5155 DEGs (2707 upregulated and 2448 downregulated genes) in the differentiated D7 sample, and 6777 DEGs (3871 upregulated and 2906 downregulated genes) in the D14 sample. The heatmap depicted in Figure 1A highlights the top 20 DEGs exhibiting either upregulation or downregulation across D2, D7, and D14 samples.

Figure 1Analysis of DEGs during the differentiation of hiPSCs into cardiomyocytes. (A) The heatmap displays the top 20 DEGs exhibiting either upregulation or downregulation in the D2 sample (A1), D7 sample (A2), and D14 sample (A3). The D0 sample indicates undifferentiated iPSCs (green box), while the D2, D7, and D14 samples correspond to mesoderm cells, early cardiomyocytes, and mature cardiomyocytes (red box), respectively. Genes are labeled on the *y*‐axis, with blue denoting downregulated genes and red indicating upregulated genes. (B) An analysis of the top 10 GO functions associated with the DEGs. (C) The KEGG pathway enrichment analysis identifies the top five KEGG signaling pathways linked to the DEGs. (D) A Venn diagram illustrates the common DEGs, with (D1) representing downregulated DEGs across the three samples and (D2) depicting upregulated DEGs. (E) A PPI network analysis of the common DEGs is shown, with red representing upregulated genes and green indicating downregulated genes. (F) The top 10 genes identified from the PPI network are illustrated; circles denote downregulated genes while diamonds represent upregulated genes. The colors red, orange, jacinth, and yellow reflect a decrease in gene connectivity.

**Figure:**
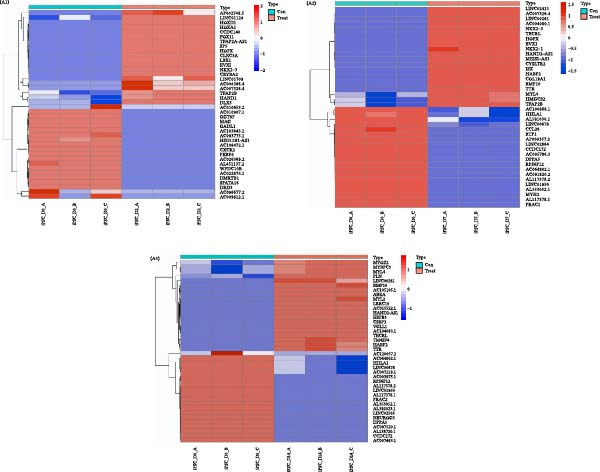
(A)

**Figure:**
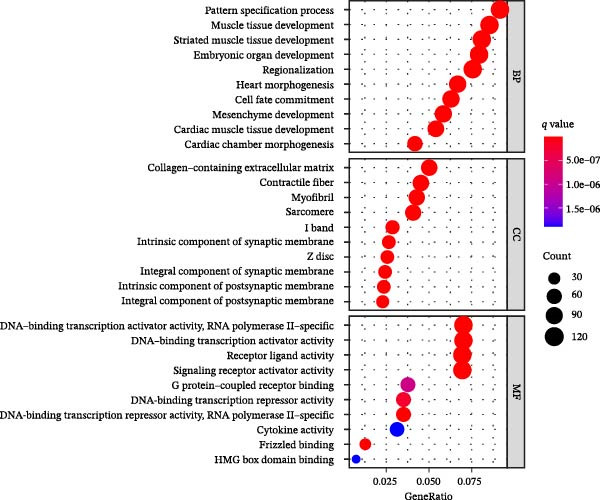
(B)

**Figure:**
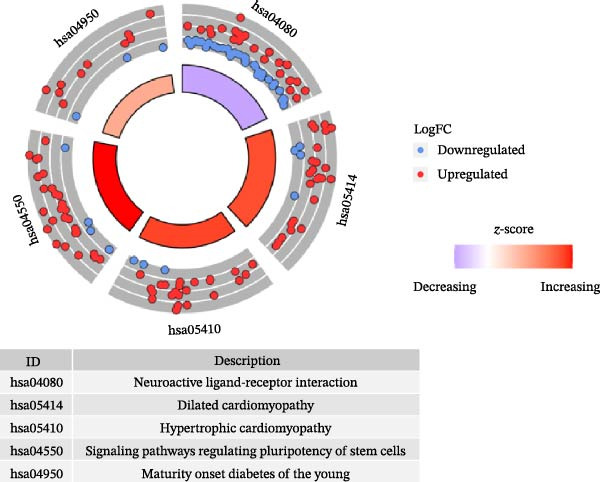
(C)

**Figure:**
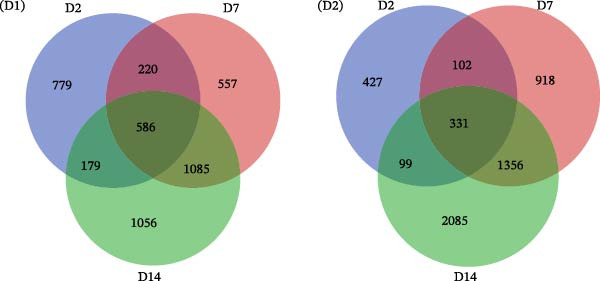
(D)

**Figure:**
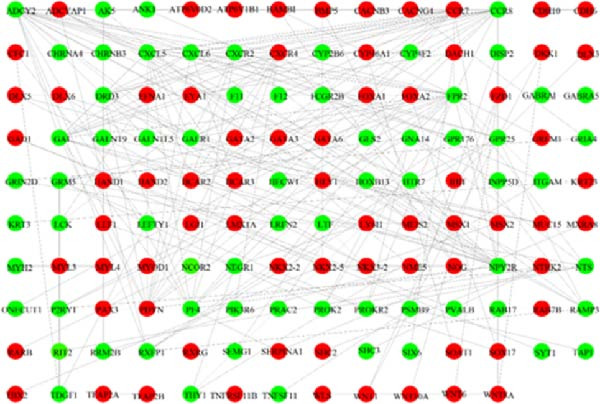
(E)

**Figure:**
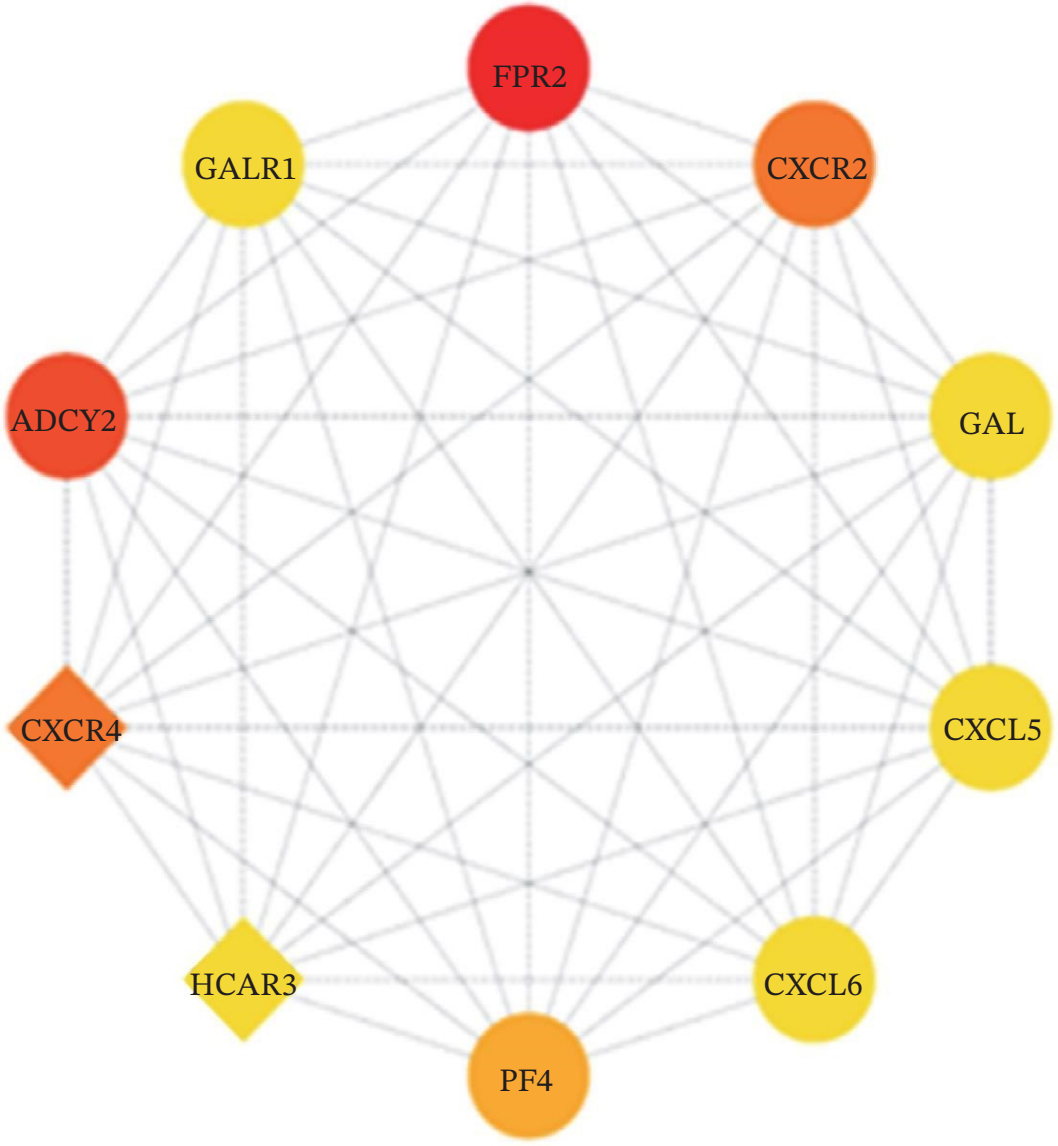
(F)

The subsequently obtained DEGs were subjected to GO functional analysis and KEGG analysis. GO functional analysis indicated that the DEGs predominantly participate in myocardial tissue development, contractile fiber formation, and G protein‐coupled receptor interactions (Figure 1B, Supporting Information 1: Figure S1). The top 10 functions of DEGs in D2 samples are shown in Supporting Information 1: Figure S1A, including embryonic organ development (GO_BP), embryonic organ morphogenesis (GO_BP), synaptic membrane (GO_CC), collagen‐containing extracellular matrix (ECM) (GO_CC), DNA‐binding transcription activator activity (GO_MF), and G protein‐coupled receptor binding (GO_MF). The top 10 functions of DEGs in D7 samples are shown in Supporting Information 1: Figure S1B, including heart morphogenesis (GO_BP), cardiac chamber morphogenesis (GO_BP), transmembrane transport complex (GO_CC), ion channel complex (GO_CC), receptor ligand activity (GO_MF), and growth factor activity (GO_MF). While the top 10 functions of DEGs in D14 samples are shown in Supporting Information 1: Figure S1C, including muscle tissue development (GO_BP), myocardial tissue development (GO_BP), contractile fibers (GO_CC), myofibrils (GO_CC), signal receptor activator activity (GO_MF), and metal ion transmembrane transporter activity (GO_MF).

In parallel, KEGG pathway analysis demonstrated that DEGs in the D2 samples were mainly enriched in neuroactive ligand‐receptor interactions, signaling pathways regulating stem cell pluripotency, and chemokine signaling pathways. In the D7 samples, the DEGs were mainly enriched in the PI3K‐Akt signaling pathway, adrenergic signaling in cardiomyocytes, and myocardial contraction. In the D14 samples, the DEGs were mainly enriched in the Ras signaling pathway, regulation of the actin cytoskeleton, and dilated cardiomyopathy (Figure 1C, Supporting Information 1: Figure S2). However, the neuroactive ligand‐receptor interactions were the most enriched signaling pathway, with 68 DEGs in the D2 sample, 113 DEGs in the D7 sample, and 138 DEGs in the D14 sample enriched in this pathway.

From the three groups, a total of 586 DEGs exhibited downregulation, while 331 DEGs were upregulated (Figure 1D). The STRING database facilitated the construction and visualization of the PPI network for the common DEGs among the three samples (Figure 1E). The top 10 genes with the highest interaction degrees within the PPI network were identified using Cytoscape: *FPR2*, *ADCY2*, *CXCR2*, *CXCR4*, *PF4*, *GALR1*, *GAL*, *CXCL5*, *CXCL6*, and *HCAR3* (Figure 1F). In this study, based on GO and KEGG analyses, *CXCR4* was found to be mainly enriched in cardiac processes, heart contraction, and regulation of growth and development, indicating that *CXCR4* plays pivotal role in the differentiation of iPSCs into cardiomyocytes.

### 3.2. Cluster Analysis of Samples and WGCNA

The gene expression profile data (GSE137920), reflecting four distinct stages of iPSC differentiation into cardiomyocytes—designated as D0, D2, D7, and D14—was analyzed using R software. Genes exhibiting expression averages surpassing the overall mean were classified as highly expressed and subsequently analyzed utilizing the WGCNA plugin (Version 1.69). A cluster analysis was performed on the expression data concerning iPSC differentiation into cardiomyocytes, as depicted in Figure 2A. Outlier samples were identified and excluded from analysis, allowing for the selection of normal samples for further examination. The determination of a suitable soft threshold for constructing scale‐free networks was achieved at a scale‐free index of network topology *R* ^2^ = 0.9, with a soft threshold *β* set at 12, resulting in an average connectivity trending towards zero (Figure 2B).

Figure 2Cluster dendrogram and separation of highly expressed genes into modules. (A) Sample cluster analysis. (B) Generation of a scale‐free fitting index of network topology via soft threshold analysis. (C) Cluster dendrogram and separation of highly expressed genes into modules. The height is the cutoff value, the cluster dendrogram is the cluster tree, the dynamic tree cut is the initial module, and the merged dynamic is the final module after the merger. (D) The numbers of genes contained in different modules. Different colors represent different modules, each leaf node in the tree diagram represents a gene, and densely connected branches represent closely related genes. (E) Interaction between genes in the green gene module (relationship of gene interactions in the green gene module; red indicates genes that interact with the most with other genes). (F) Venn diagram analysis of differentially expressed genes and important modules.

**Figure:**
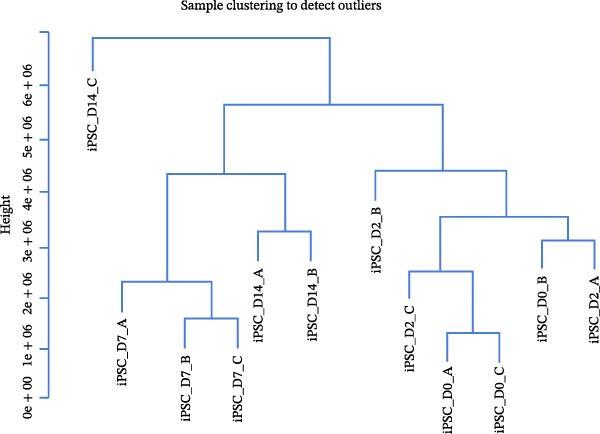
(A)

**Figure:**
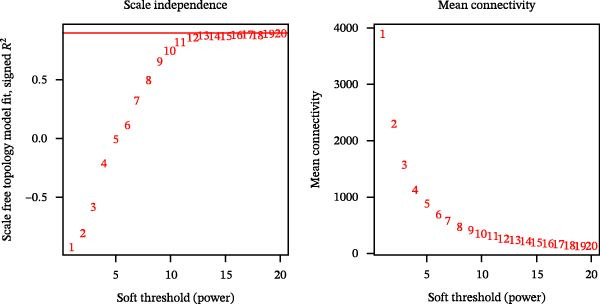
(B)

**Figure:**
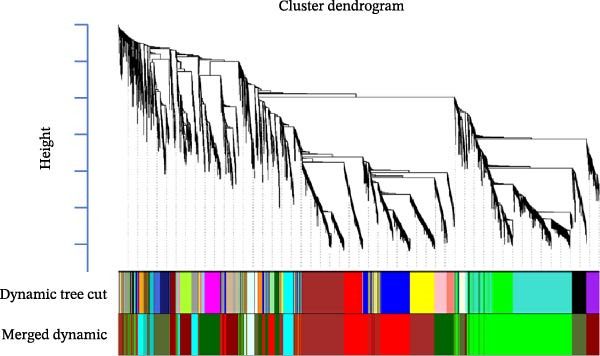
(C)

**Figure:**
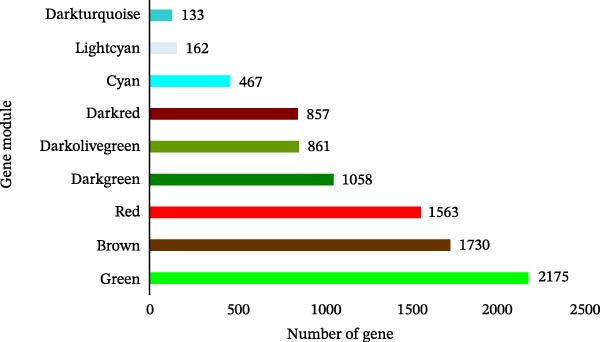
(D)

**Figure:**
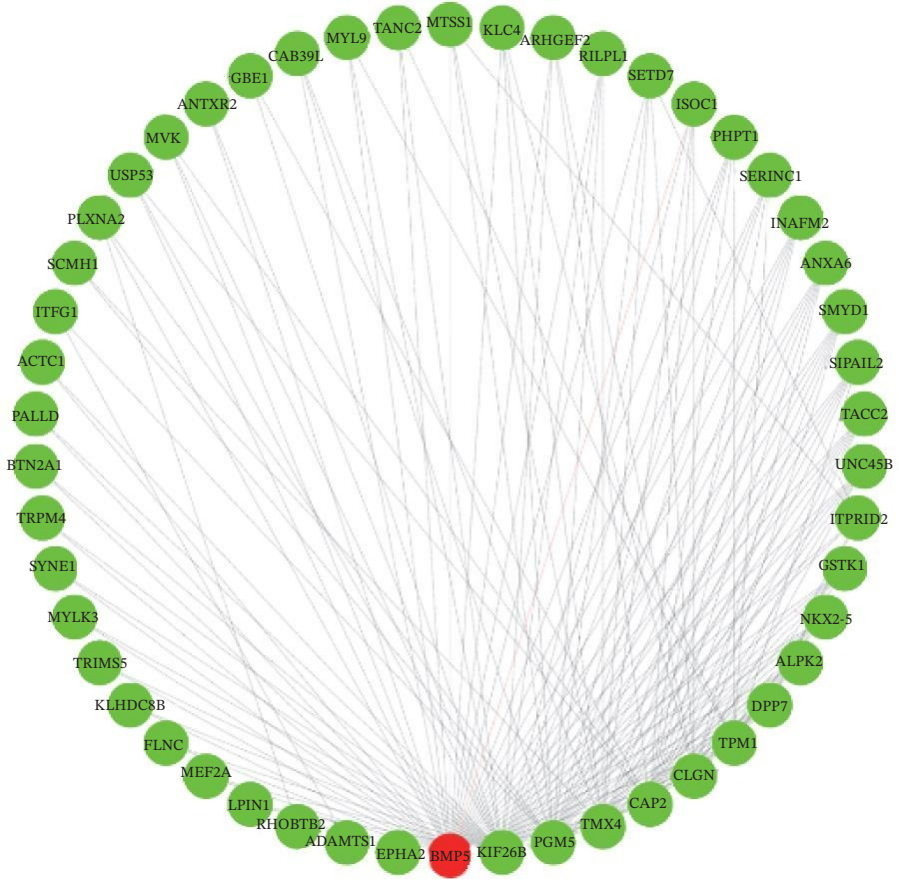
(E)

**Figure:**
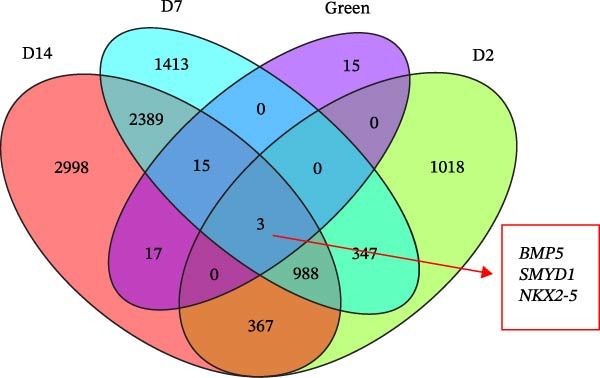
(F)

Following the filtering process, genes demonstrating low expression across all samples were discarded, culminating in the selection of 9006 genes for WGCNA analysis. Merging of modules with a similarity indicator exceeding 0.75 yielded a total of 9 gene modules (Figure 2C). The composition of each module, as illustrated in Figure 2D and Supporting Information 1: Table S1, indicates the number of genes contained within. A heatmap was subsequently constructed to evaluate the relationships between modules and their associated traits, revealing a significant clustering of the green module, which encompassed 2175 genes.

### 3.3. Gene Interactions and Screening of Hub Genes in the Module

WGCNA was applied to analyze regulatory networks between genes in modules and identify hub genes. In the green module, 399 interacting genes were screened (weight >0.695). The top 50 genes that interacted the most with each other in the module were visualized, and the gene that interacted with the most other genes was BMP5 (Figure 2E). These 50 genes were subsequently combined with the DEGs in the D2, D7, and D14 samples to construct Venn diagrams, and three co‐expressed genes, namely, *BMP5*, *SMYD1*, and *NKX2-5*, were identified (Figure 2F). The STRING database was used to construct a PPI network for these three genes, and *BMP5* and *SMYD1* were found to interact with *NKX2-5* (Supporting Information 1: Figure S3). Among them, BMP5 was not only expressed in the D2, D7, and D14 samples but also interacted with other genes that were the most common in the green gene module, indicating that *BMP5* is a hub gene in the module and plays an important role in inducing the differentiation of iPSCs into cardiomyocytes.

### 3.4. Induction of hESC Differentiation Into Cardiomyocytes and Detection of ALP Activity

The hESCs were grown on the basement membrane matrix Geltrex in PGM1 PSC medium as a feeder‐free monolayer (Figure 3A). A small area of pulsatile cells could be observed by microscopy on Day 6. The presence of pulsatile cells indicated that the hESC had differentiated into cardiomyocytes. On the 14th day, a large area of pulsatile cells appeared (Figure 3B).

Figure 3Induction hESC differentiation into cardiomyocytes. (A) The differentiation process of hESCs into cardiomyocytes. (B) Cell morphology of hESCs differentiation into cardiomyocytes. (C) Alkaline phosphatase detection during differentiation of hESCs into cardiomyocytes (200 µm).  ^∗∗^ *p* < 0.01,  ^∗∗∗^ *p* < 0.001, compared with the control group.

**Figure:**
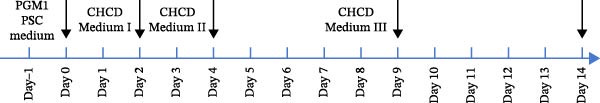
(A)

**Figure:**
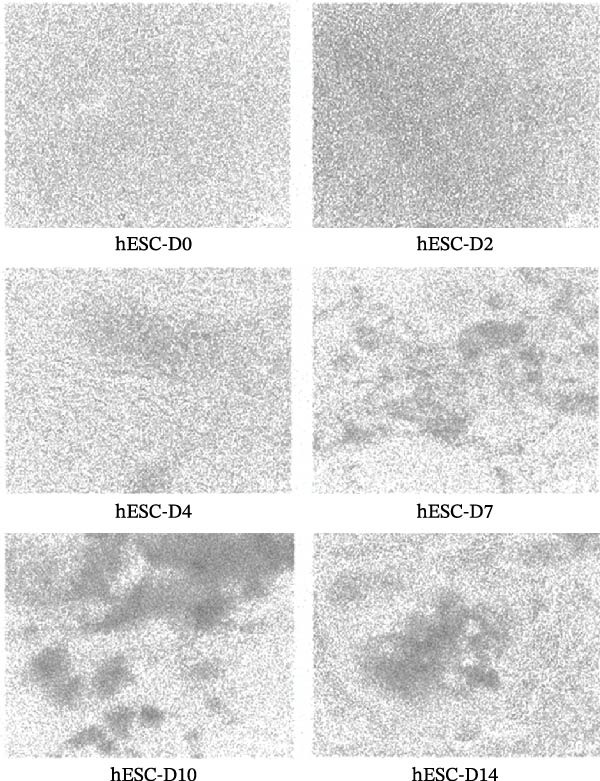
(B)

**Figure:**
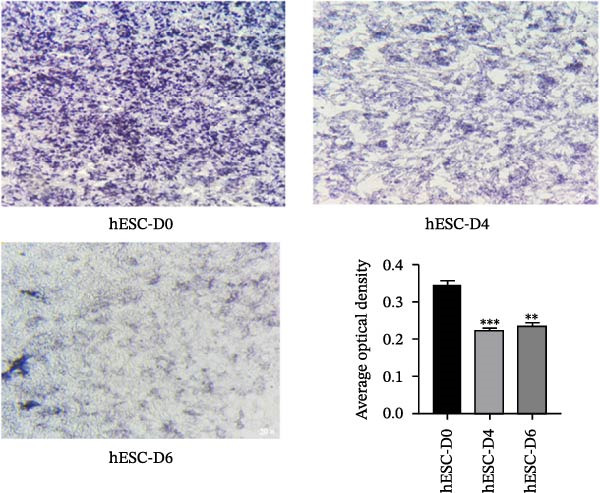
(C)

To detect changes in the expression of marker molecules during cell differentiation, immunohistochemistry staining of ALP was used. ESCs displayed positive ALP activity, which is a phenotypic marker of ESCs. As shown in Figure 3C, ALP staining significantly decreased on the 4th and 6th days of differentiation into cardiomyocytes compared with that in undifferentiated hESCs (Day 0).

### 3.5. Expression of Stage‐Specific Markers During ESC Differentiation Into Cardiomyocyte

The expression levels of stage‐specific marker genes were assessed in cells differentiated from hESCs at Days 0 (hESC‐D0), 2 (hESC‐D2), 7 (hESC‐D7), and 14 (hESC‐D14). Compared with that in hESC‐D0, the expression of the mesoderm marker gene MESP1 significantly increased in hESC‐D2 and remained high in hESC‐D7 and hESC‐D14. The early cardiac‐specific markers NKX2−5 and ISL1 presented increasing expression levels starting from hESC‐D2, with sustained elevation in hESC‐D7 and hESC‐D14. The cardiac‐specific marker cTnT was significantly increased in hESC‐D7 and hESC‐D14. Conversely, the expression of the pluripotency markers SOX2 and C‐MYC notably decreased as differentiation progressed (Figure 4A). Consequently, during the cell differentiation process, hESC‐D2 represents the mesoderm developmental stage, hESC‐D7 signifies the early cardiac cell developmental stage, and hESCs‐D14 corresponds to the cardiac cell developmental stage.

Figure 4Identification of specific markers of hESC differentiation into cardiomyocytes. (A) Expression of marker genes, including *MESP1*, *NKX2-5*, *ISL1*, *cTnT*, *SOX2*, and *C-MYC*, at each stage of hESC differentiation into cardiomyocytes. (B) Expression of key genes at each stage of hESC differentiation into cardiomyocytes. (C) Image of a beating hESC‐derived cardiomyocyte mass (Supporting Information 2: Video S1). (D) The expression of the cardiomyocyte marker protein α‐actinin was detected by western blotting. (E) The expression of the cardiomyocyte marker protein cTnT was detected by immunofluorescence.  ^∗^ *p* < 0.05,  ^∗∗^ *p* < 0.01,  ^∗∗∗^ *p* < 0.001,  ^∗∗∗∗^ *p* < 0.0001, with the control group.

**Figure:**
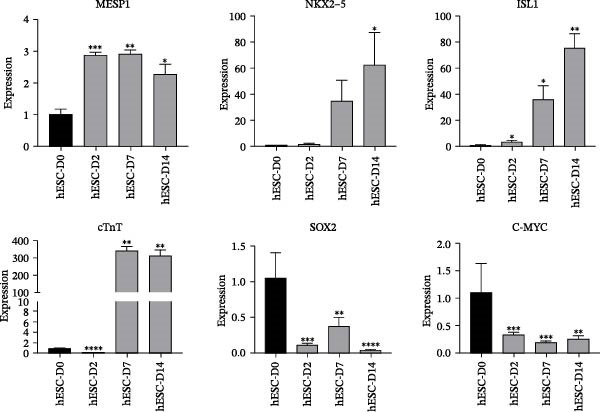
(A)

**Figure:**
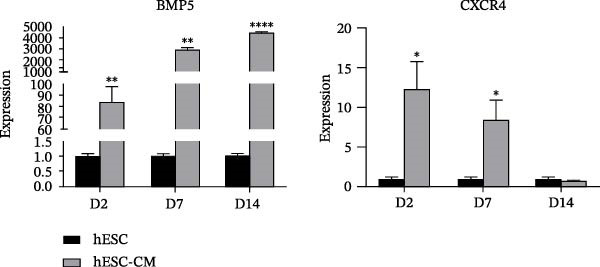
(B)

**Figure:**
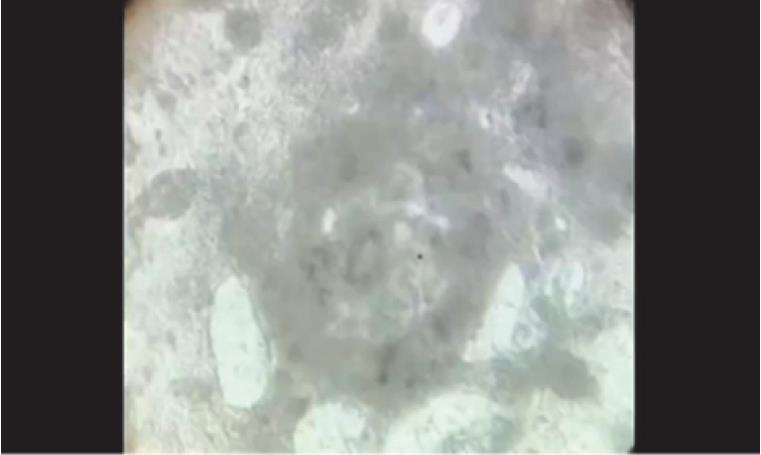
(C)

**Figure:**
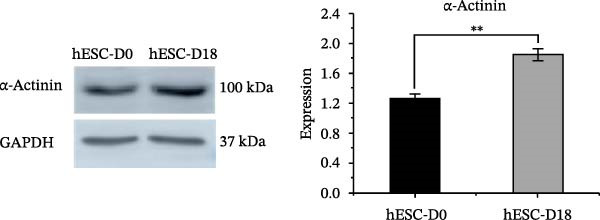
(D)

**Figure:**
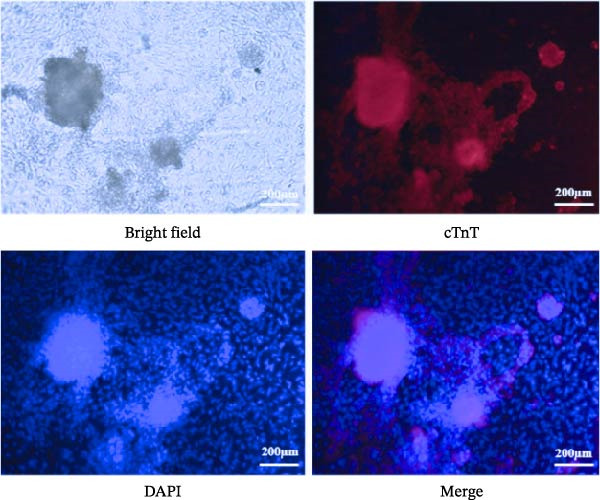
(E)

Preliminary analysis revealed that BMP5 and CXCR4 might divert hPSC to cardiomyocyte differentiation. Therefore, real‐time PCR was used to determine the expression of *CXCR4* and *BMP5* at different stages of cardiomyocyte differentiation. Compared with that in hESCs, the expression levels of BMP5 in hESC‐D2, hESC‐D7, and hESC‐D14 were significantly elevated. However, *CXCR4* exhibited increased expression levels in hESC‐D2 and hESC‐D7, with no significant change in expression in hESC‐D14 (Figure 4B).

On Day 8 of induced differentiation, cardiomyocytes derived from BMP5‐GFP plasmid‐transfected ESCs began to exhibit pulsatile cell clumps (Figure 4C, Supporting Information 2: Video S1). The expressions of the cardiomyocyte marker proteins cardiac troponin (cTnT) and α‐actinin were detected by immunofluorescence and western blotting. Western blotting indicated that the expression of α‐actinin was increased significantly after 18 days of differentiation compared with that in hESCs (Day 0) (Figure 4D). cTnT is located only on cardiomyocytes and plays an important role in regulating their contraction. cTnT was detected in the 18th day of hESC differentiation into cardiomyocytes by immunofluorescence (Figure 4E). Moreover, hESC‐derived cardiomyocytes were able to beat. The above experiments confirmed that we successfully induced the differentiation of hESCs into cardiomyocytes.

### 3.6. Effects of BMP5 Overexpression on the Proliferative Capacity and Pluripotency of hESCs

To investigate the impact of BMP5 overexpression on hESCs, we conducted a transfection of hESCs with the engineered Ad‐BMP5‐EGFP vector. Following a 72‐h incubation period, we confirmed the successful introduction of the GFP, as evidenced by its expression (Figure 5A). Subsequently, we aimed to determine whether BMP5 overexpression influences the pluripotent characteristics of hESCs. We employed immunofluorescence to assess the expression levels of pluripotency markers TRA‐1‐60 and TRA‐1‐81 in both BMP5‐transfected and nontransfected hESCs, all cultured for a duration of 72 h. The findings, displayed in Figure 5B, indicated that both TRA‐1‐60 and TRA‐1‐81 were present in nontransfected hESCs as well as in BMP5‐transfected hESCs, with no significant differences observed between the two groups. This suggests that the overexpression of BMP5 does not adversely affect the pluripotency of hESCs.

Figure 5Effect of *BMP5* overexpression on the proliferation and pluripotency of hESCs. (A) Expression of green fluorescence observed at 48 h (A1) and 72 h (A2) posttransfection. (B) Immunofluorescence analysis of pluripotency markers TRA‐1‐60 and TRA‐1‐81 in BMP5‐transfected versus nontransfected hESC populations (200 µm). (C) ELISA quantification of BMP5 levels in culture supernatants collected at 72 and 96 h. (D) CCK‐8 assay results measuring hESC proliferation at 48 and 72 h.  ^∗^ *p* < 0.05, compared with the control group.

**Figure:**
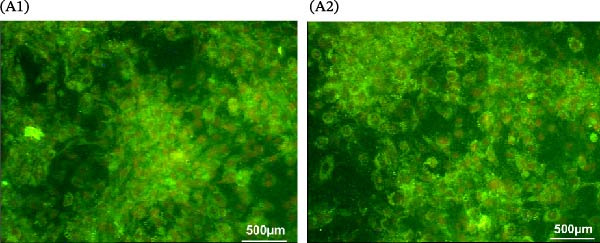
(A)

**Figure:**
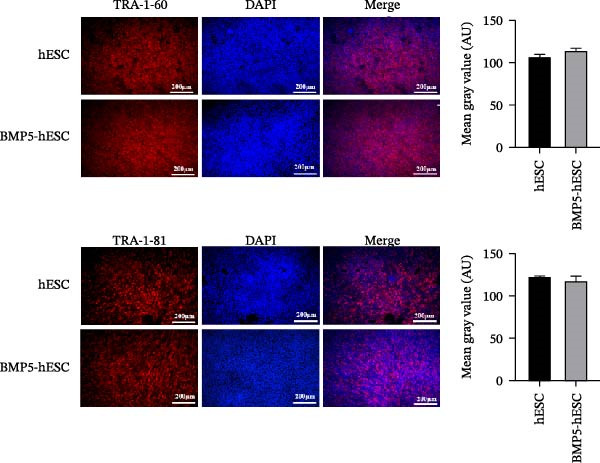
(B)

**Figure:**
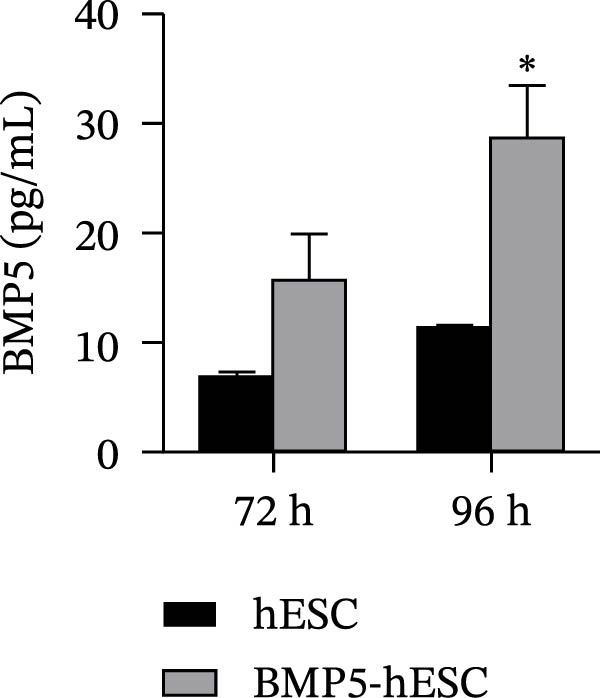
(C)

**Figure:**
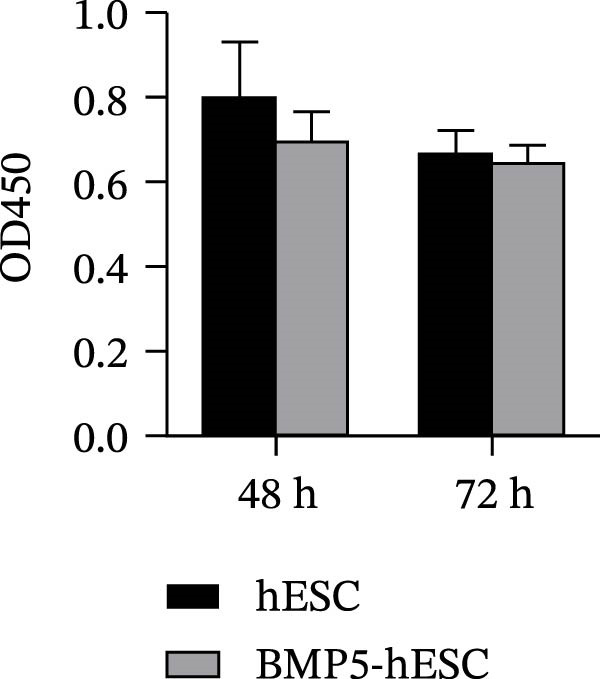
(D)

Given that BMP5 is a secreted protein, we proceeded to collect the culture supernatants from the transfected cells after 72 and 96 h for analysis via ELISA. The results demonstrated that BMP5 levels in the supernatants from the transfected groups were significantly elevated compared to those in the control group, confirming the high efficiency of transfection and enabling us to advance to differentiation induction experiments (Figure 5C). We then explored whether BMP5 overexpression impacted the proliferation rates of hESCs. The proliferation of both BMP5‐transfected and nontransfected hESCs was quantified using the CCK‐8 assay at 48‐ and 72‐h posttransfection. As illustrated in Figure 5D, there was no significant difference in the proliferation capacity between the BMP5‐transfected and nontransfected groups, indicating that *BMP5* overexpression did not influence the proliferative potential of hESCs, allowing for further exploration into differentiation towards cardiomyocytes.

### 3.7. Overexpression of BMP5 Promotes hESCs Differentiation Into Cardiomyocytes

In the subsequent phase of our study, we assessed the ability of BMP5 to facilitate the differentiation of hESCs into cardiomyocytes. Following a 72‐h culture period, both Ad‐BMP5‐EGFP‐transfected and nontransfected hESCs were subjected to cardiomyocyte differentiation medium. The differentiation timeline is depicted in Figure 6A. By Day 8 of differentiation, observable pulsatile cell clumps emerged in the BMP5‐transfected cardiomyocytes (BMP5‐hESC‐CM) at a frequency of ~28 beats per minute, while the nontransfected hESC‐derived cardiomyocytes (hESC‐CM) showed no such pulsatile activity. By Day 9, pulsatile cell clusters were evident in the nontransfected group, albeit at a lower frequency of ~23 beats per minute. Throughout the period from Day 8 to Day 14, the transfected group exhibited a significantly higher number of beats compared to the nontransfected counterparts (Figure 6B).

Figure 6Differentiation process and effects of BMP5 overexpression on the expression of marker proteins in hESC‐derived cardiomyocytes. (A) Differentiation process of BMP5‐GFP plasmid‐transfected of hESCs into cardiomyocytes. (B) Measurement of the pulsatile frequency in hESC‐derived cardiomyocytes. (C) On Day 14 of differentiation, immunofluorescence techniques were employed to assess the expression levels of cardiac troponin cTnT. (D) To evaluate the expression of marker proteins cTnT and α‐actinin, western blotting was conducted on samples collected on the 12th day of the differentiation protocol.  ^∗^ *p* < 0.05,  ^∗∗∗^ *p* < 0.01, compared with the control group.

**Figure:**
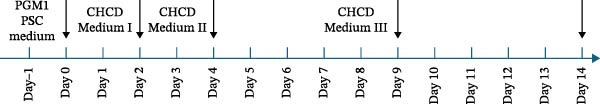
(A)

**Figure:**
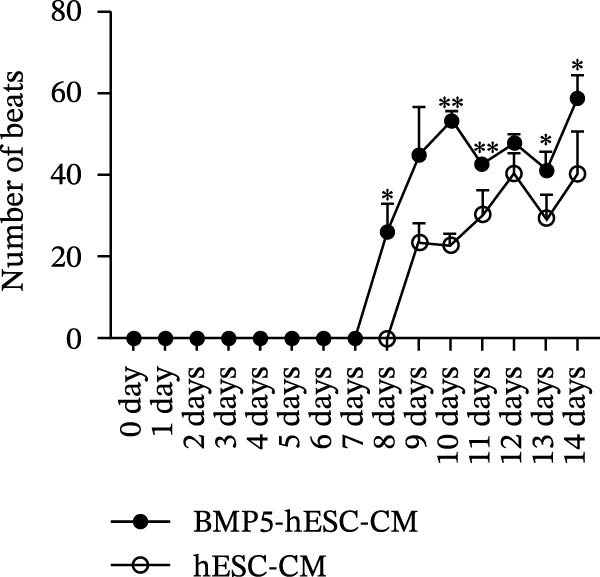
(B)

**Figure:**
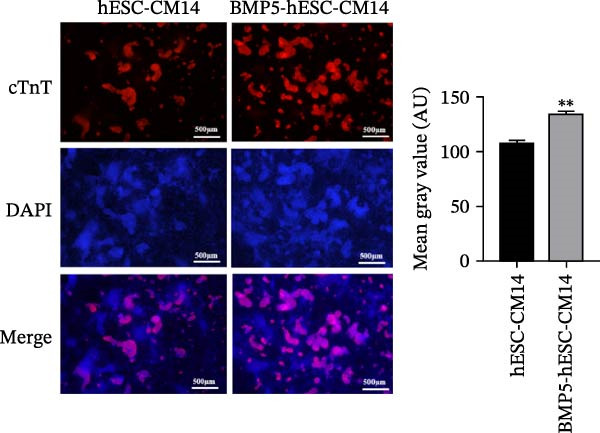
(C)

**Figure:**
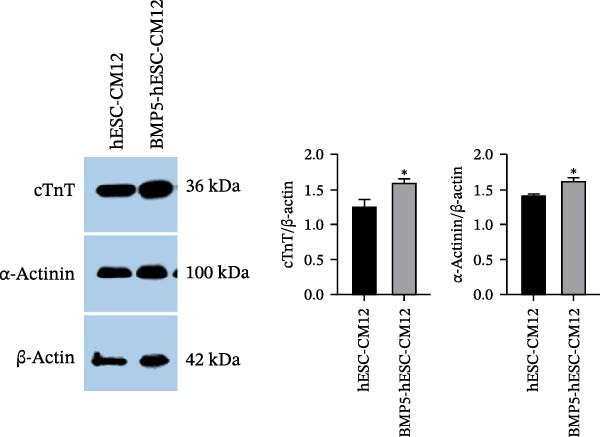
(D)

On Day 14 of the differentiation process, we utilized immunofluorescence to investigate the expression of the cardiomyocyte‐specific marker cTnT. This analysis revealed the presence of cTnT‐positive cell clusters in both the transfected (BMP5‐hESC‐CM14) and nontransfected (hESC‐CM14) groups; however, the transfected group exhibited a markedly higher quantity of cTnT‐positive clusters (illustrated in Figure 6C). Additionally, western blot analysis performed on Day 12 of differentiation indicated that the transfected group displayed elevated levels of both cTnT and α‐actinin, further confirming the enhanced cardiomyocyte differentiation resulting from BMP5 overexpression (Figure 6D).

### 3.8. The Effect of BMP5‐KO on the Differentiation of hESCs Into Cardiomyocytes

Subsequently, BMP5 was knocked out from hESCs using CRISPR/Cas9 genome editing technology to explore the biological role of BMP5 in hESCs (Supporting Information 1: Figures S4 and S5). After culturing both BMP5‐KO hESCs and control hESCs in cardiomyocyte differentiation medium for a duration of 10 days, distinct pulsatile cell aggregates emerged in the control group, while no pulsatile cell clusters were observed in the BMP5‐KO group. Transcriptomic analysis indicated a pronounced clustering trend between the BMP5‐KO and control groups (Figure 7A, Supporting Information 1: Table S3). A total of 2259 DEGs were identified, with 1897 exhibiting significant upregulation and 362 showing significant downregulation. Notably, genes associated with myocardial developmental regulation, such as *ACTC1*, *GRCM1*, *CXCL8*, *HAND1*, *ANKRD1*, *CDH6*, *ZIC1*, *HAPLN1*, *NPPB*, and *MYL4*, were significantly downregulated. Conversely, genes implicated in embryonic development and cellular differentiation, including *POSIN*, *FMOD*, *ITIH5*, *CPXM2*, *PRSS35*, *SHISA3*, *CAPN6*, *DCN*, and *ITGA10*, were found to be significantly upregulated.

Figure 7Transcriptomic analysis comparing the BMP5 knockout group to the control group. (A) A volcanic map illustrating the distribution of differential gene expression, where the scattered dots represent individual genes—gray dots indicate genes without significant differences, red dots denote significantly upregulated genes, and blue dots represent significantly downregulated genes. (B) GO functional enrichment analysis of DEGs between the BMP5 knockout and control groups. (C) KEGG pathway enrichment analysis of DEGs between BMP5 knockout and control groups. (D) Construction of the PPI network.

**Figure:**
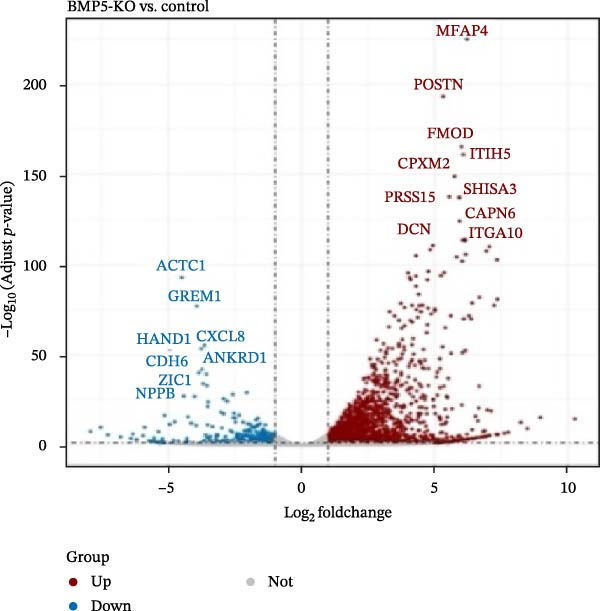
(A)

**Figure:**
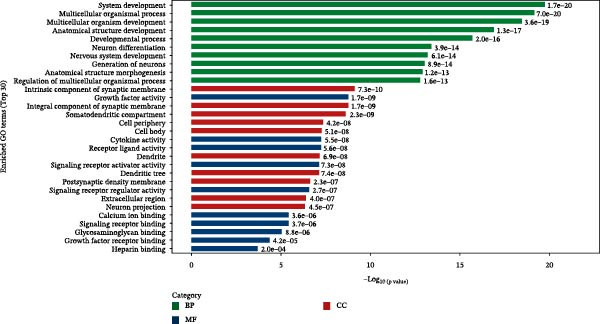
(B)

**Figure:**
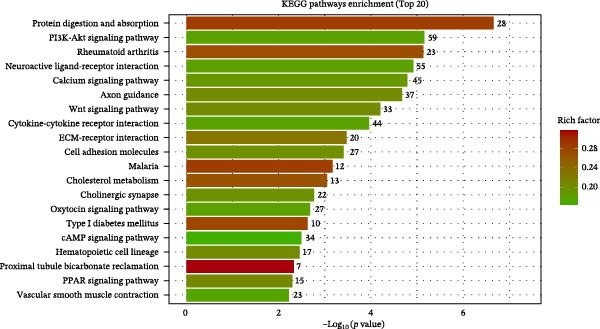
(C)

**Figure:**
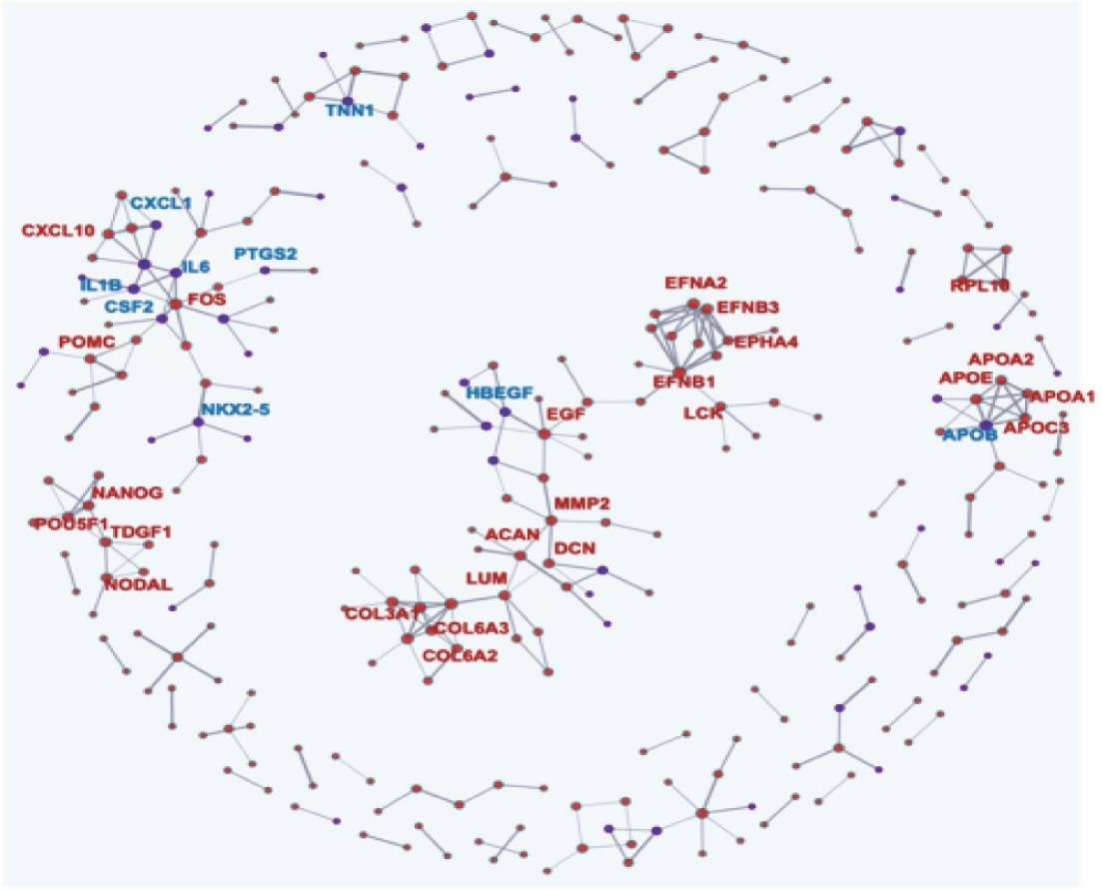
(D)

The results of GO analysis vividly illustrated the functional enrichment characteristics of all DEGs, highlighting significant enrichment in genes related to essential biological processes (Figures S6–S8). The top 30 GO terms for downregulated genes underscored that BMP5‐KO significantly influenced various biological processes (Figure 7B), including system development, multicellular organismal processes, organismal development, anatomical structure development, and general developmental processes.

Furthermore, the KEGG pathway enrichment analysis of DEGs associated with metabolic pathways indicated that BMP5‐KO markedly affected the differentiation of ESCs in various cellular processes, including substance transport and metabolism (Figure 7C). The analysis revealed that DEGs were predominantly enriched in several pathways, particularly those related to protein digestion and absorption, the PI3K‐Akt signaling pathway, rheumatoid arthritis, neuroactive ligand‐receptor interactions, and calcium signaling pathways. The top 20 pathways identified through KEGG analysis included various downregulated pathways pertinent to myocardial development, such as the calcium signaling pathway, Wnt signaling pathway, cytokine–cytokine receptor interactions, cell adhesion molecules, cAMP signaling pathway, and vascular smooth muscle contraction.

Subsequently, a PPI network was used for identification of pivotal genes. The results showed that BMP knockout had a significant impact on the expression of genes associated with pluripotency transcription factors, including NANOG, POU5F1, TDGF1, and NODAL. Additionally, genes related to the ECM, particularly those in the collagen subfamily (*COL3A1*, *COL6A2*, *COL6A3*, and *LUM*) and the apolipoprotein subfamily (*APOA1*, *APOA2*, and *APOB3*, *APOE*), were markedly upregulated following BMP5‐KO. Conversely, genes implicated in cardiac development, such as *NKX2-5* and *TNN1*, as well as immune regulation genes (*IL-6*, *IL1B*, *PTGS2*, and *CSF2*) and *HBEGF*, showed a significant decrease in expression after BMP5‐KO. Therefore, the bioinformatics outcomes derived from transcriptome sequencing indicate that the knockout of the BMP5 gene may inhibit the expression of genes associated with cardiomyocyte development and their related signaling pathways.

## 4. Discussion and Conclusion

The transforming growth factor‐beta (TGF‐β) superfamily comprises more than 15 known BMPs that share structural similarities. BMPs are categorized into subgroups based on amino acid homology, including BMP2/4, BMP5/6/7/8, BMP9/BMP10, and BMP12/13/14 (GDF5/6/7) [12]. These proteins are crucial for embryogenesis, particularly during the stages of embryonic development, and play a role in maintaining homeostasis within adult tissues. BMP signaling is fundamental during early developmental processes, facilitating cell growth, apoptosis, and differentiation [13].

BMPs have a close association with endocardial formation and differentiation, demonstrating the capacity to induce embryonic myocardial formation and differentiation through the initiation of mCherryNTR expression and the recombinant human myosin protein light chain‐7 [14, 15]. During mesoderm formation and heart development, the knockout of BMP2 or BMP4 leads to a deceleration in the endocardium‐to‐mesenchyme transition, resulting in severe defects in endocardial formation and subsequent embryonic lethality [16–18]. Furthermore, BMP2 and BMP4 can promote the differentiation of stem cells into cardiomyocytes [19, 20]. Homozygous mutants for BMP2 are associated with embryonic lethality [16]. Together with BMP2, BMP4 is essential for the development of the atrioventricular septum of the heart [21]. The signaling pathway of BMP4 from the myocardium to the endocardium is integral to this process, and its conditional inactivation is linked to atrioventricular canal defects (AVCDs) [22]. In comparison to control mice, BMP10 knockout mice showed a marked reduction in cardiomyocyte proliferation. BMP10 is prominently expressed in the heart during both development and postnatal stages, playing a pivotal role in cardiac formation [10, 23].

RNA sequencing (RNA‐seq) identified downregulated DEGs in the BMP5‐KO group, the majority of which are crucial for myocardial development or associated with myocardial diseases. Notable genes exhibiting significantly decreased expression include *ACTC1*, *GREM1*, *HAND1*, and *MYL4*. The ACTC1 protein is primarily involved in the formation and maintenance of the cytoskeleton during cardiac muscle contraction and is extensively distributed throughout the ventricles and atria, contributing to the proper contraction and relaxation of the heart [22]. Mutations and aberrant expression of ACTC1 have been linked to various cardiovascular diseases and cardiomyopathies, underscoring the importance of studying its structure and function for biomedical applications [23, 24]. Gremlin‐1 (GREM1), a member of the BMP antagonist family, is known to be associated with cell proliferation and survival [25]. Elevated levels of GREM1 have been shown to enhance the survival capacity of aged cardiac mesenchymal progenitor cells through the upregulation of the ERK/NRF2‐associated antioxidant signaling pathway [26]. HAND1 is critical for cardiac development, particularly during the differentiation of cardiomyocytes [27]. Research indicates that the expression of the HAND1 gene is diminished in the hearts of patients with ischemic myocardial conditions (myocardial infarction), indicating its significant involvement in sustaining cardiac function [28]. In the early stages of human development, HAND1 is responsible for regulating the differentiation of epicardial mesenchymal cells (ExMC) by interacting with ape‐specific long terminal repeat (LTR) sequences, thereby contributing to the establishment of a microenvironment conducive to pluripotency maintenance [13]. The MYL4 protein demonstrates multiple functions within cardiomyocytes [29]. It serves as a distinctive marker for Purkinje fibers (PFs) in the human heart, facilitating the localization and visualization of the PF distribution network. Research has indicated that MYL4 exhibits high expression levels inPFs, with its protein uniformly disseminated throughout the cell, which surpasses the membrane‐restricted expression observed with the conventional marker GJA5/Cx40 [30]. BMP5, a vital component of the BMP family, plays a crucial role in various biological processes, including cell proliferation, apoptosis, chondrogenesis, stem cell differentiation, human embryonic development, tissue and organ repair and growth, as well as tumorigenesis and prognosis [31–35]. BMP5 has been shown to facilitate the differentiation of mesenchymal stem cells into cardiomyocytes in murine models [36, 37]. Recent studies have reported that inhibition of BMP1.3 reduces scar formation, supports cardiomyocyte survival after myocardial infarction, and induces cardioprotection through BMP5 [38]. Testing under hypoxic conditions to rescue cardiomyocyte survival showed that BMP5 alone or in combination with BMP2 exerted the highest cardioprotective effect. When expression was inhibited using siRNA, the absence of BMP5 was observed to eliminate the protective activity of the anti‐BMP1.3 antibody. Overall, these findings suggest that BMP5 mediates the early cardioprotective effects of the anti‐BMP1.3 antibody and that BMP5 is a key mediator of cardioprotection [38]. Nonetheless, the specific role of BMP5 in the differentiation of hPSCs into cardiomyocytes remains to be elucidated.

Currently, research focusing on the molecular aspects of each stage of hPSCs differentiation into cardiomyocytes is limited, with a notable lack of systematic screening and analysis of critical genes involved in the entire differentiation process. In this investigation, we employed differential gene expression analysis alongside WGCNA to identify key genes and the molecular regulatory networks implicated in the differentiation of hPSCs into cardiomyocytes and to evaluate the biological significance of these key genes. The functional role of the selected key gene, BMP5, was examined through experimental approaches. The findings suggest that BMP5 may be pivotal in the differentiation of hPSCs into cardiomyocytes [39].

BMP5 was primarily enriched in pathways associated with myocardial tissue development, embryonic organ growth, and cardiac morphogenesis. Not only did BMP5 interact with other genes within the essential gene module of iPSC differentiation, but it also exhibited significant expression across three stages of iPSC differentiation into cardiomyocytes, namely mesodermal cells, early cardiomyocytes, and mature cardiomyocytes. The interaction between BMP5 and NKX2−5, a crucial transcription factor involved in cardiac development, was confirmed through PPI network analysis. Consequently, we propose that BMP5 is integral to the differentiation of iPSCs into cardiomyocytes.

In this study, following the transfection of hESCs with a BMP5‐expression plasmid and subsequent induction of differentiation into cardiomyocytes, we observed that the beating frequency and expression levels of cardiomyocyte marker proteins in cells derived from BMP5‐transfected ESCs surpassed those in cells from nontransfected ESCs. Conversely, the knockout of BMP5 in ESCs prior to differentiation induction resulted in transcriptome sequencing revealing a marked decrease in the expression of genes pertinent to myocardial development. Hence, we postulate that BMP5 may facilitate the differentiation of pluripotent stem cells into cardiomyocytes.

## Author Contributions

Conceptualization was performed by Xinjian Qu and Simei Tu. Methodology was developed by Hong Zhang and Simei Tu. Software implementation was carried out by Chang Xu. Validation was conducted by Chang Xu, Hong Zhang, and Ruoshi Wu. Formal analysis was executed by Ruoshi Wu. Investigation was led by Xinjian Qu. Resources were managed by Simei Tu and Xinjian Qu. Data curation was handled by Jiayi Wu and Xin Huang. The original draft was prepared by Xinjian Qu, Simei Tu, and Hong Zhang. Review and editing of the manuscript were undertaken by Xiaowei Luo and Xinjian Qu. Visualization was accomplished by Hong Zhang. Supervision was provided by Xiaowei Luo and Xinjian Qu. Project administration was overseen by Xiaoqin Luo and Xinjian Qu. Funding acquisition was secured by Xinjian Qu.

## Funding

This research was funded by the Natural Science Foundation of Guangxi (Grant 2024GXNSFAA010459) and the Research Launching Fund Project from Guangxi University of Chinese Medicine Introduced Doctoral (Grant 2022BS021).

## Disclosure

This work was performed at Guangxi University of Chinese Medicine, Institute of Marine Drugs, Nanning, China. All authors provided their final approval and accepted accountability for all aspects of the work. The authors take full responsibility for the final content of the article.

## Ethics Statement

The study received approval from the Ethics Committee of Guangxi University of Chinese Medicine (Number GXTCMU ‐ EC20250805‐174).

## Consent

The authors have nothing to report.

## Conflicts of Interest

The authors declare no conflicts of interest.

## Supporting Information

Additional supporting information can be found online in the Supporting Information section.

## Supporting information


**Supporting Information 1** Figure S1: Analysis of top 10 GO functions of DEGs in D2, D7 and D14 samples; Figure S2: KEGG analysis of DEGs in D2, D7 and D14 samples; Figure S3: Analysis of key genes in the differentiation of iPSCs into cardiomyocytes. Figure S4: Schematic diagram of human BMP5 knockout model using CRISPR/Cas‐mediated lentiviral vector for genome engineering. Figure S5: Western blot to detect the BMP5 expression in BMP5 knockout hESCs group and control group. Figure S6: GSEA was conducted using GO pathways’ biological process branch as the gene sets of interest.; Figure S7: GSEA was conducted using GO pathways’ cellular component branch as the gene sets of interest; Figure S8: GSEA was conducted using GO pathways’ molecular function branch as the gene sets of interest. Table S1: The number of genes contained in different modules. Table S2. Important pathway enrichment analysis; Table S3: Transcriptome sequencing analysis of DEGs in BPM5‐KO.

**Supporting Information 2** Video S1: Video of pulsatile cell lumps.

## Data Availability

The data supporting the findings of this study can be accessed from the corresponding author upon reasonable request.
