# Supplementary material for: Screening and Identification of BMP5 as a Key Regulatory Gene for hPSCs Transcardiomyocyte Differentiation
Source: Stem Cells Int. 2026 Apr 24;2026:5540587. doi: 10.1155/sci/5540587 (PMC13108435; doi:10.1155/sci/5540587)
Supplement: Supplementary file 1 — Supporting Information 1 Figure S1: Analysis of top 10 GO functions of DEGs in D2, D7 and D14 samples; Figure S2: KEGG analysis of DEGs in D2, D7 and D14 samples; Figure S3: Analysis of key genes in the differentiation of iPSCs into cardiomyocytes. Figure S4: Schematic diagram of human BMP5 knockout model using CRISPR/Cas‐mediated lentiviral vector for genome engineering. Figure S5: Western blot to detect the BMP5 expression in BMP5 knockout hESCs group and control group. Figure S6: GSEA was conducted using GO pathways’ biological process branch as the gene sets of interest.; Figure S7: GSEA was conducted using GO pathways’ cellular component branch as the gene sets of interest; Figure S8: GSEA was conducted using GO pathways’ molecular function branch as the gene sets of interest. Table S1: The number of genes contained in different modules. Table S2. Important pathway enrichment analysis; Table S3: Transcriptome sequencing analysis of DEGs in BPM5‐KO. [file SCI-2026-5540587-s002.zip › Suporting information/Additional file 1∩╝êFigure S1-S8+Table S1-S2).pdf]

Screening and identification of BMP5 as a key regulatory gene for hPSC transcardiomyocyte differentiation

Hong Zhang. ***et al.***

List of Supporting Information

Figure S1. Analysis of top 10 GO functions of DEGs in D2, D7 and D14 samples.
Figure S2. KEGG analysis of DEGs in D2, D7 and D14 samples.
Figure S3. Analysis of key genes in the differentiation of iPSCs into cardiomyocytes.
Figure S4. Schematic diagram of human BMP5 knockout model using CRISPR/Cas-mediated lentiviral vector for genome engineering.
Figure S5. Western blot to detect the BMP5 expression in BMP5 knockout hESCs group and control group.
Figure S6. GSEA was conducted using GO pathways' biological process branch as the gene sets of interest.
Figure S7. GSEA was conducted using GO pathways' cellular component branch as the gene sets of interest.
Figure S8. GSEA was conducted using GO pathways' molecular function branch as the gene sets of interest.
Table S1. The number of genes contained in different modules.
Table S2. Important pathway enrichment analysis.

A

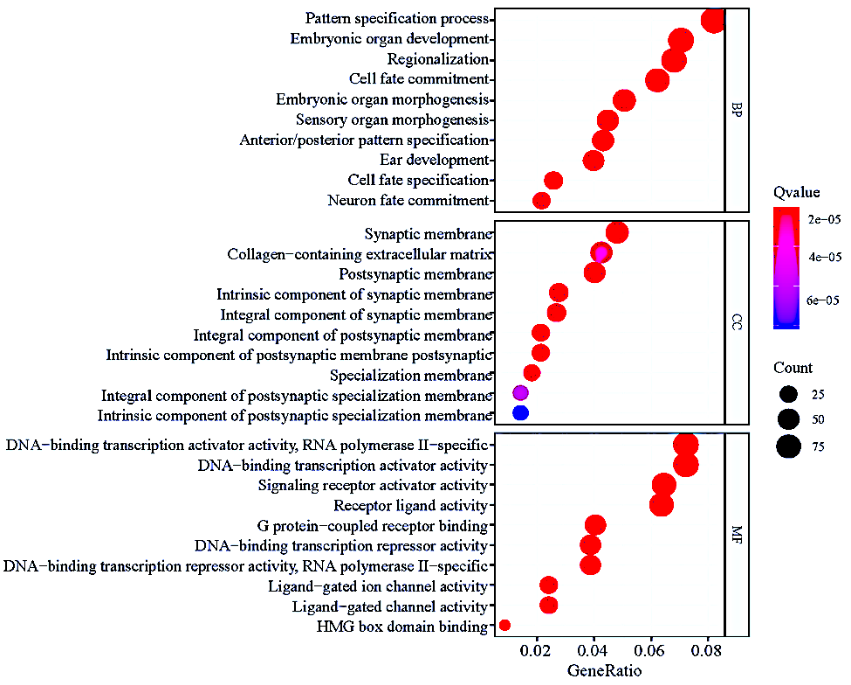

Pattern specification process
Embryonic organ development
Regionalization
Cell fate commitment
Embryonic organ morphogenesis
Sensory organ morphogenesis
Anterior/posterior pattern specification
Ear development
Cell fate specification
Neuron fate commitment
Synaptic membrane
Collagen−containing extracellular matrix
Postsynaptic membrane
Intrinsic component of synaptic membrane
Integral component of synaptic membrane
Integral component of postsynaptic membrane
Intrinsic component of postsynaptic membrane postsynaptic
Specialization membrane
Integral component of postsynaptic specialization membrane
Intrinsic component of postsynaptic specialization membrane
DNA−binding transcription activator activity, RNA polymerase II−specific
DNA−binding transcription activator activity
Signaling receptor activator activity
Receptor ligand activity
G protein−coupled receptor binding
DNA−binding transcription repressor activity
DNA−binding transcription repressor activity, RNA polymerase II−specific
Ligand−gated ion channel activity
Ligand−gated channel activity
HMG box domain binding
BP
CC
MF
0.02 0.04 0.06 0.08
GeneRatio
Qvalue
2e−05
4e−05
6e−05
Count
25
50
75

B

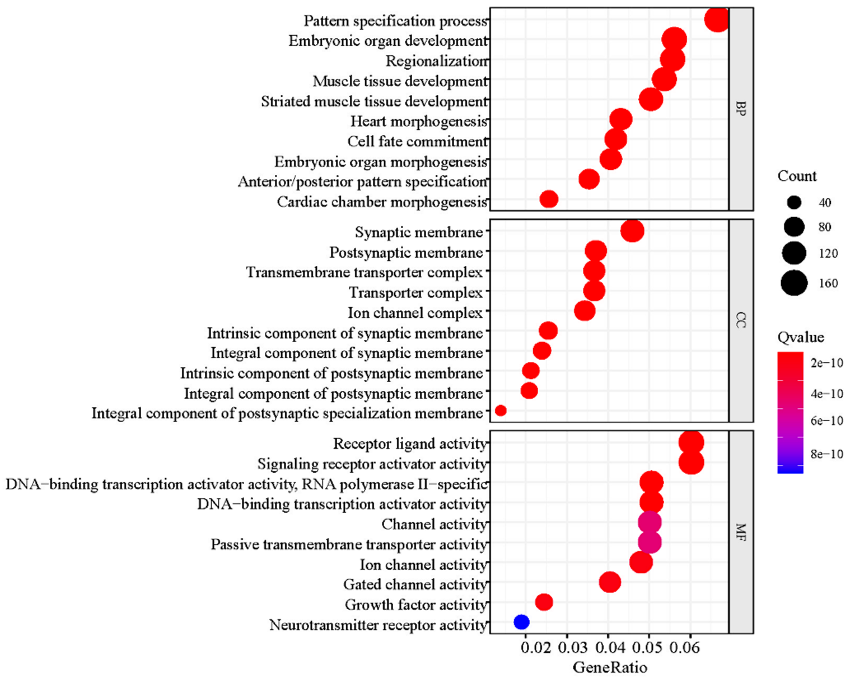

Pattern specification process
Embryonic organ development
Regionalization
Muscle tissue development
Striated muscle tissue development
Heart morphogenesis
Cell fate commitment
Embryonic organ morphogenesis
Anterior/posterior pattern specification
Cardiac chamber morphogenesis
Synaptic membrane
Postsynaptic membrane
Transmembrane transporter complex
Transporter complex
Ion channel complex
Intrinsic component of synaptic membrane
Integral component of synaptic membrane
Intrinsic component of postsynaptic membrane
Integral component of postsynaptic membrane
Integral component of postsynaptic specialization membrane
Receptor ligand activity
Signaling receptor activator activity
DNA−binding transcription activator activity, RNA polymerase II−specific
DNA−binding transcription activator activity
Channel activity
Passive transmembrane transporter activity
Ion channel activity
Gated channel activity
Growth factor activity
Neurotransmitter receptor activity
BP
CC
MF
0.02 0.03 0.04 0.05 0.06
GeneRatio
Count
40
80
120
160
Qvalue
2e−10
4e−10
6e−10
8e−10

C

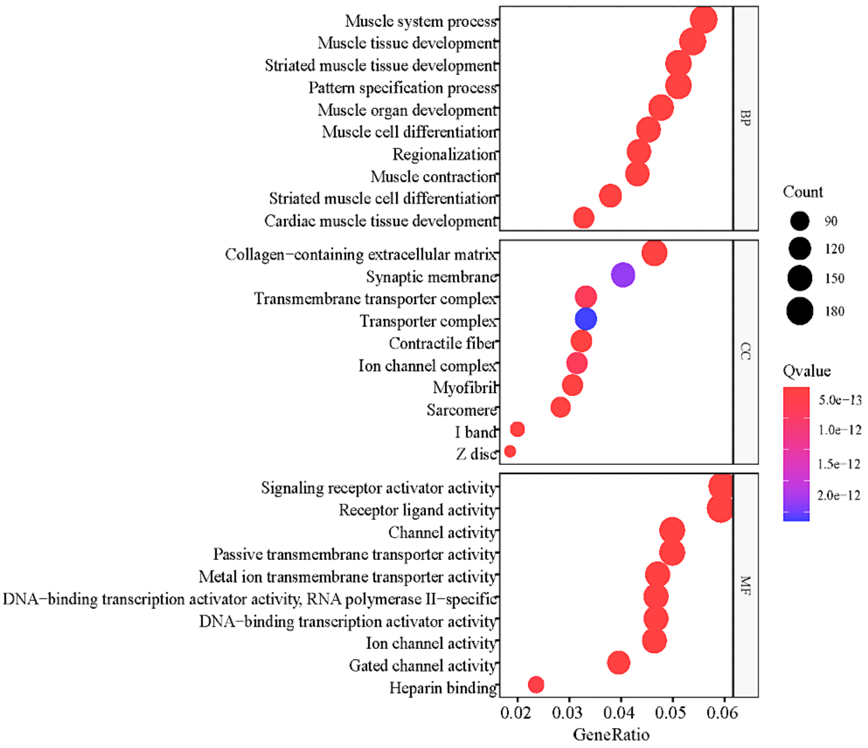

Figure S1. Analysis of top 10 GO functions of DEGs in D2, D7 and D14 samples.
(A) GO function analysis of DEGs in D2 samples. (B) GO function analysis of DEGs in D7 samples. (C) GO function analysis of DEGsin D14 samples. Q value represents the p-value corrected by the FDR method.

A

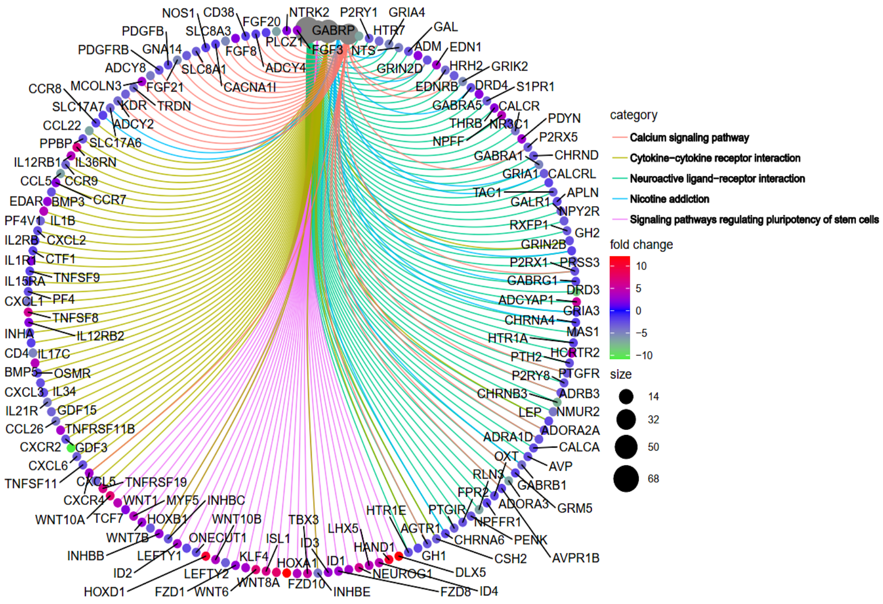

B

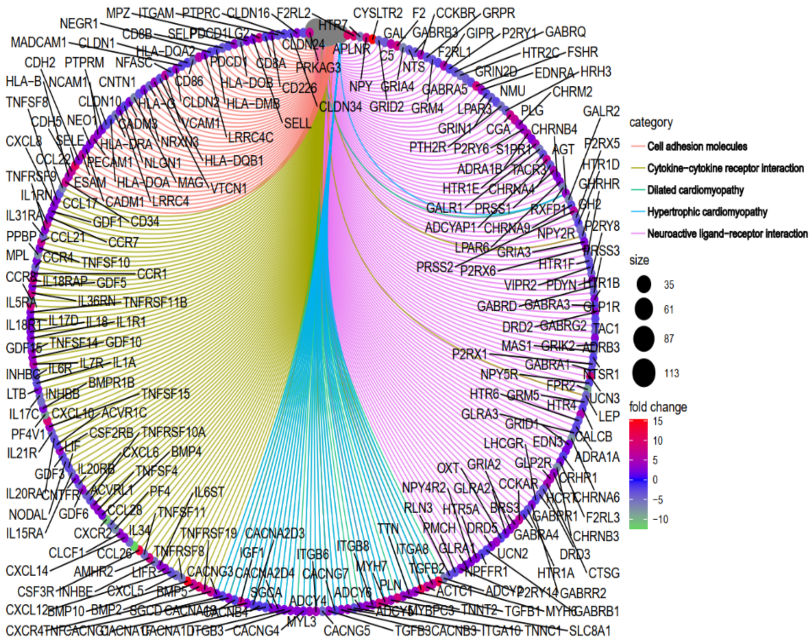

C

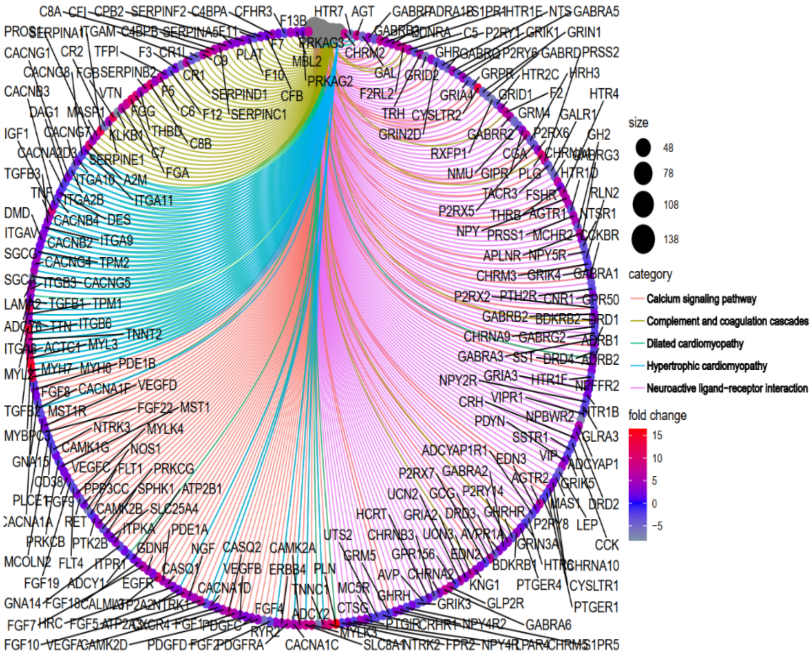

Figure S2. KEGG analysis of DEGs in D2, D7 and D14 samples.

(A) KEGG analysis of DEGs in D2 samples. (B) KEGG analysis of DEGs in D7 samples. (C)KEGG analysis of DEGs in D14 samples.

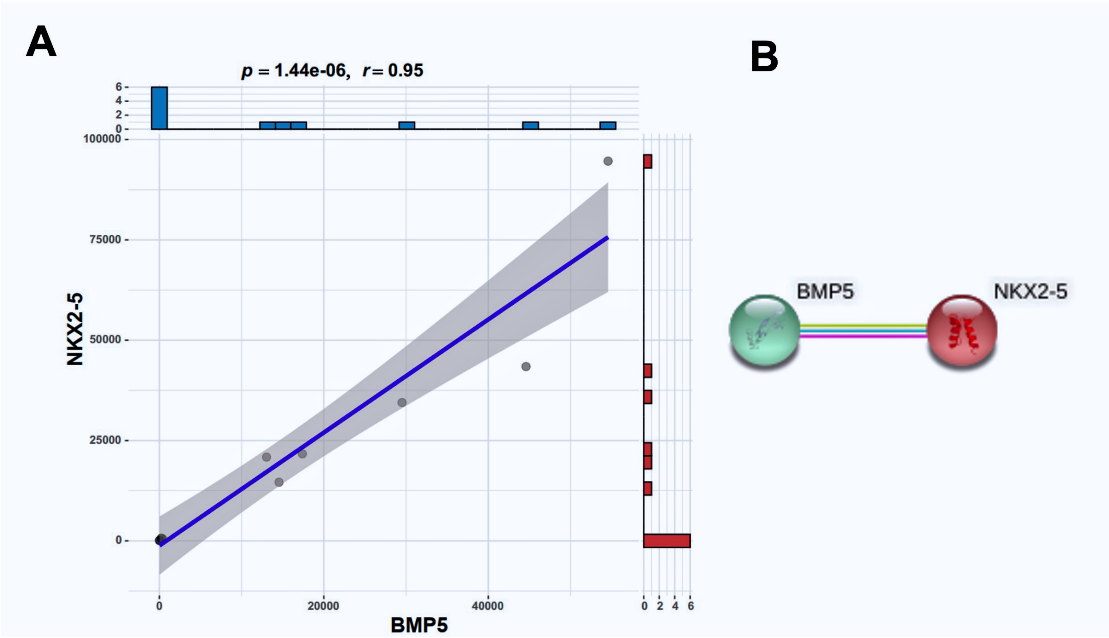

Figure S3. Analysis of key genes in the differentiation of iPSCs into cardiomyocytes. (A) Correlation analysis of NKX2-5 and BMP5. (B) PPI analysis of NKX2-5 and BMP5.

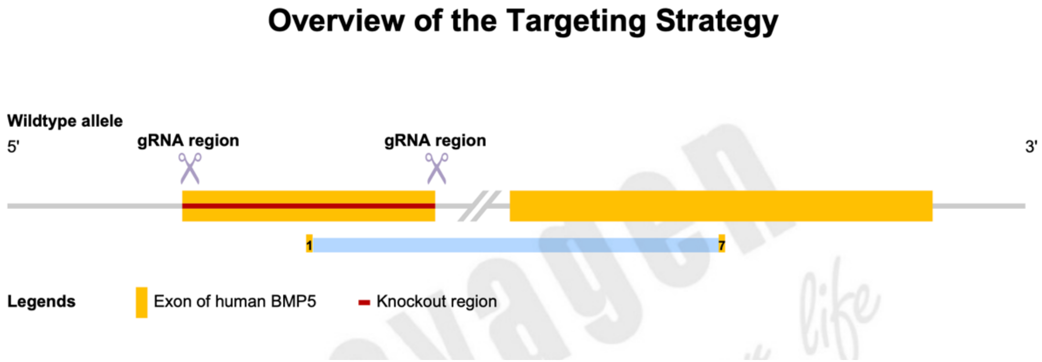

Figure S4. Schematic diagram of human BMP5 knockout model using CRISPR/Cas-mediated lentiviral vector for genome engineering.

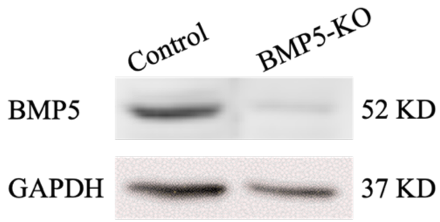

Figure S5. Western blot to detect the BMP5 expression in BMP5-KO group and control group.

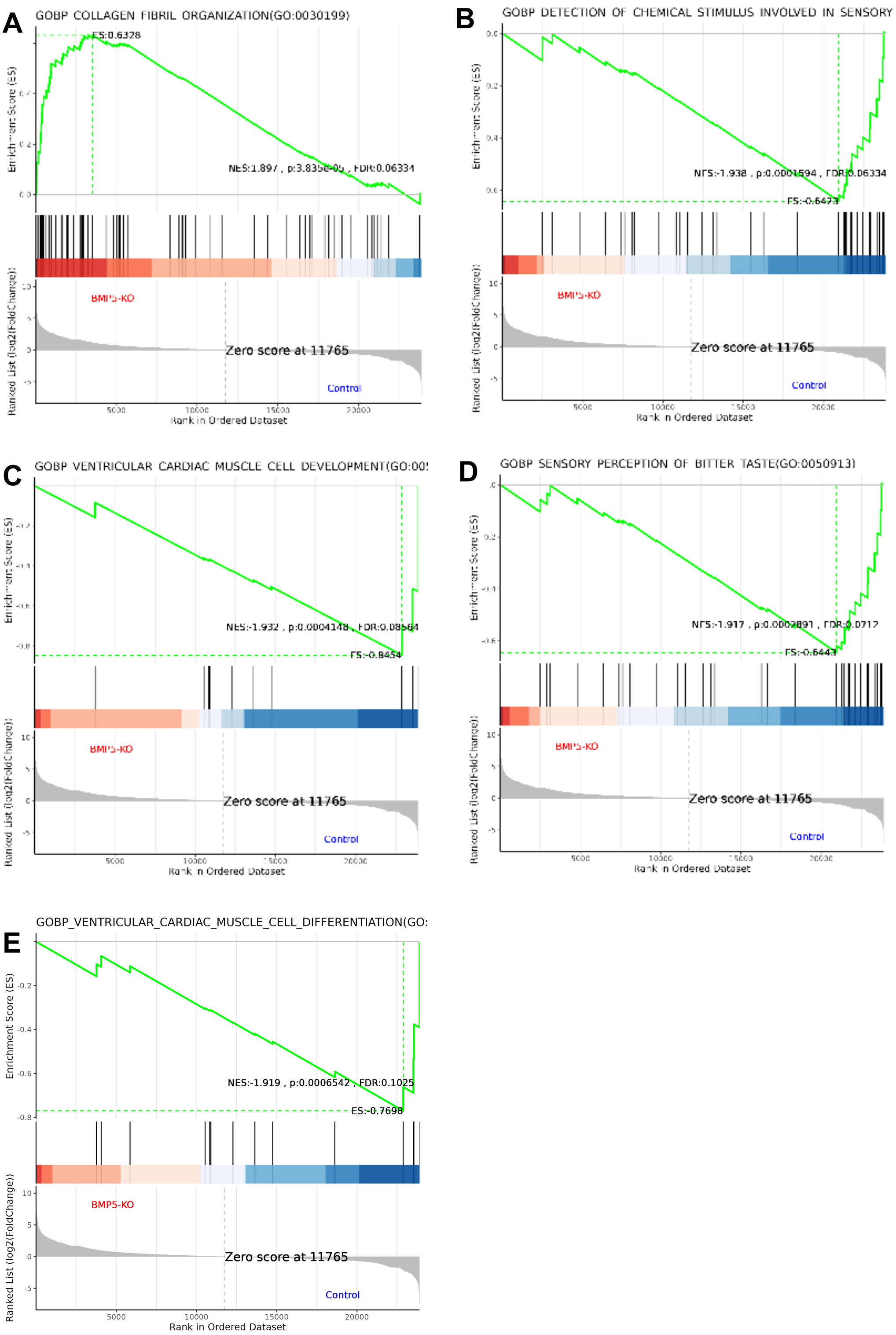

Figure S6. GSEA was conducted using GO pathways' biological process branch as the gene sets of interest.

(A) Collagen fibril organization. (B) Detection of chemical stimulus involved in sensory perception of taste. (C) Ventricular cardiac muscle cell development. (D) Sensory perception of bitter taste. (E) Ventricular cardiac muscle cell differentiation.

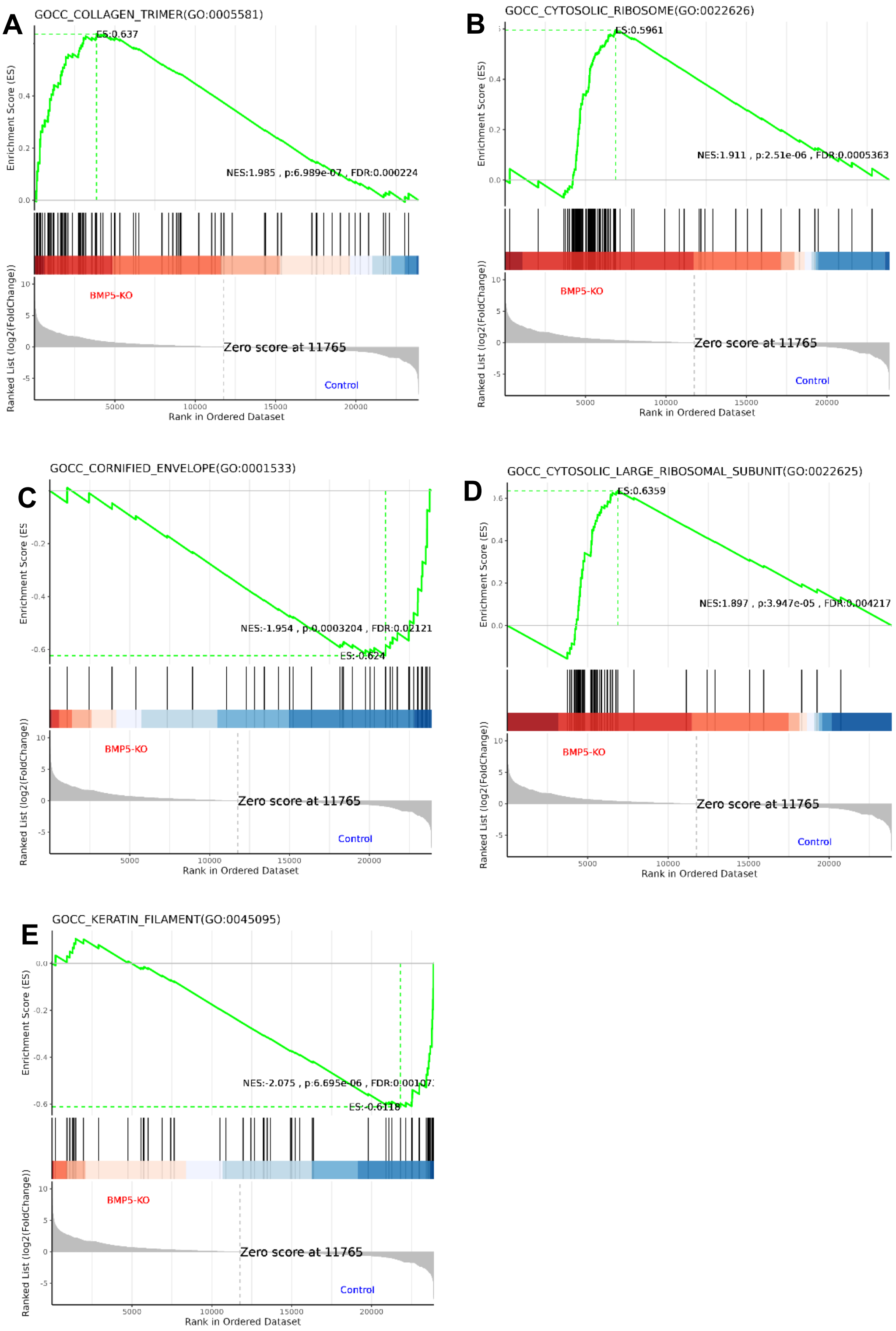

Figure S7. GSEA was conducted using GO pathways' cellular component branch as the gene sets of interest.

(A) Collagen trimer. (B) Cytosolic ribosome. (C) Cornified envelope. (D) Cytosolic large ribosomal subunit. (E) Keratin filament..

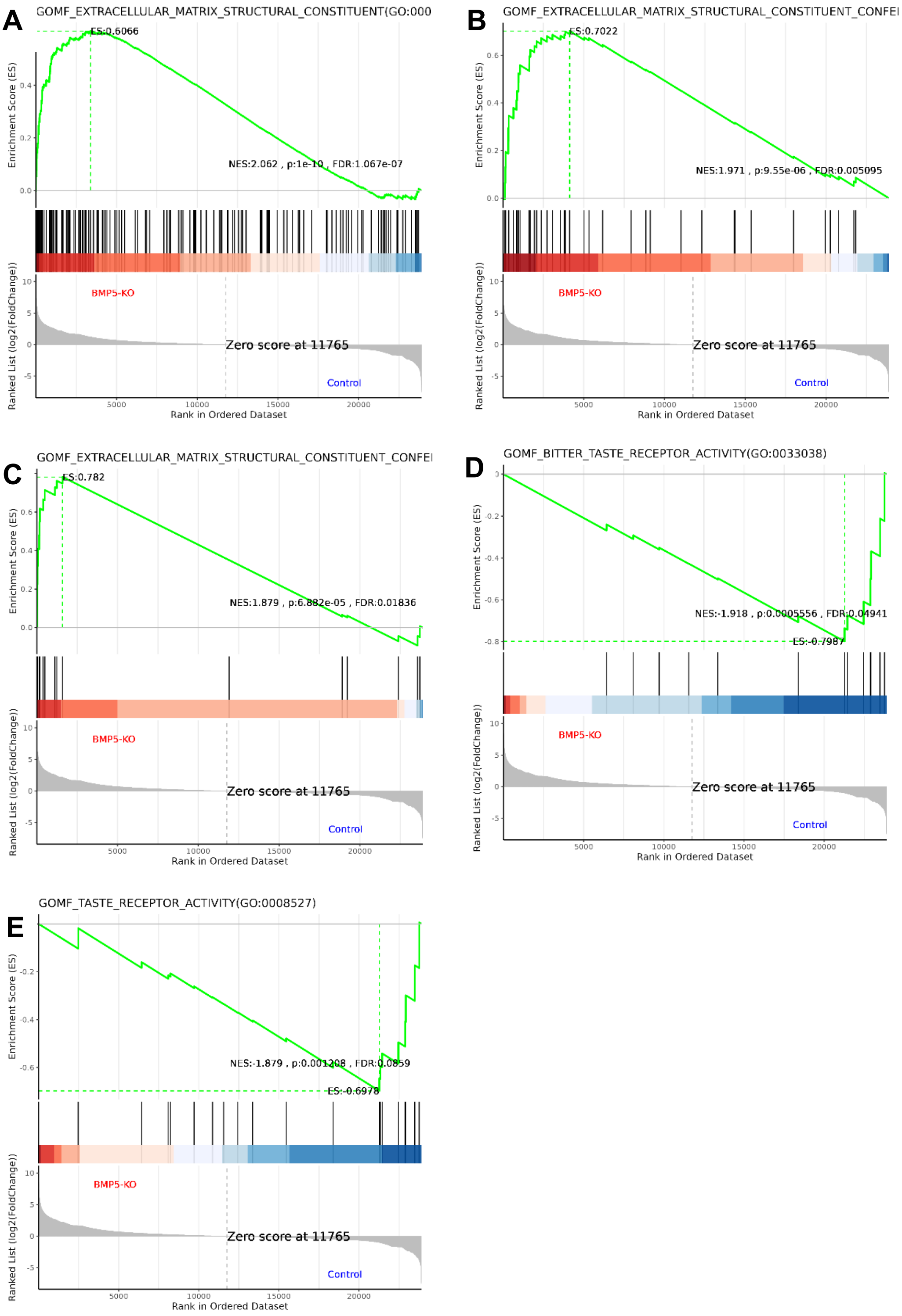

Figure S8. GSEA was conducted using GO pathways' molecular function branch as the gene sets of interest.

(A） Extracellular matrix structural constituent. (B) Extracellular matrix structural constituent conferring tensile strength. (C) Extracellular matrix structural constituent conferring compression resistance. (D) Bitter taste receptor activity. (E) Taste receptor activity.

Table S1. The number of genes contained in different modules

Gene module	Number of gene
Green	2175
Brown	1730
Red	1563
Darkgreen	1058
Darkolivegreen	861
Darkred	857
Cyan	467
Lightcyan	162
Darkturquoise	133

Table S2. Important pathway enrichment analysis

Items	Pathways	*P*-value	FDR
Green module	Post-translational protein modification	4.11E-08	5.34E-06
	Membrane trafficking	1.11E-16	4.83E-14
	Metabolism of lipids	1.64E-04	6.39E-03
	Asparagine N-linked glycosylation	1.11E-16	4.83E-14
	Cellular responses to stress	1.20E-03	3.36E-02
