# Supplementary figures and images for: Screening and Identification of BMP5 as a Key Regulatory Gene for hPSCs Transcardiomyocyte Differentiation
Source: Stem Cells Int. 2026 Apr 24;2026:5540587. doi: 10.1155/sci/5540587 (PMC13108435; doi:10.1155/sci/5540587)

**Fig. 4-D**

**$\alpha$ -actinin**

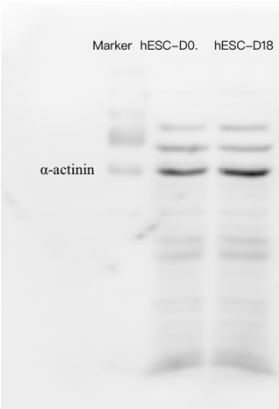

**GAPDH**

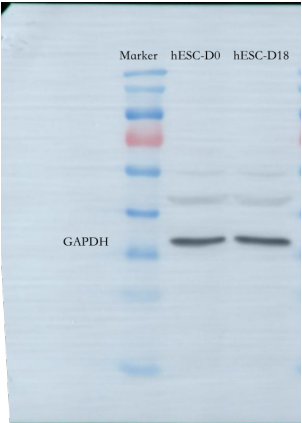

**Fig. 6-E**

**cTNT**

**$\alpha$ -actinin**

**B-actin**

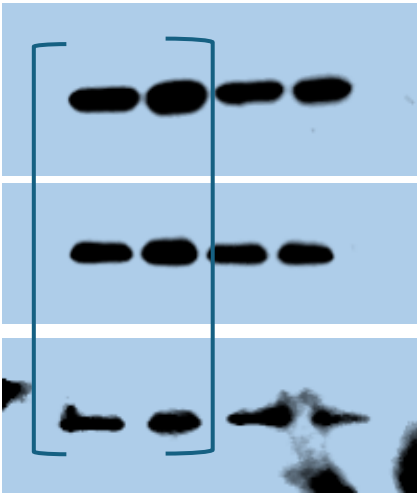

**Figure S4.**

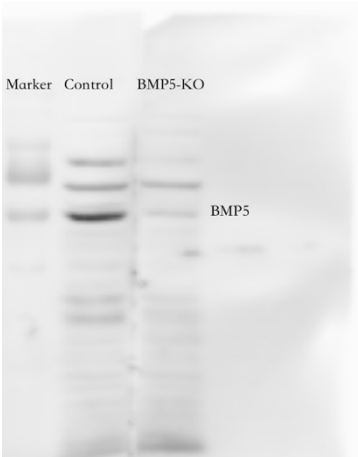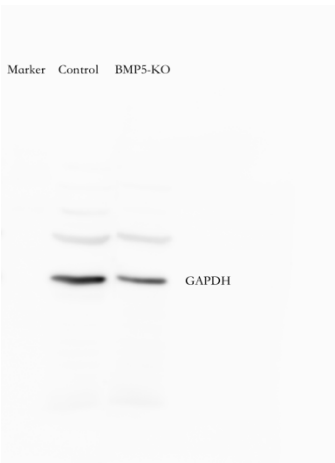

Supplement: Supplementary file 1 — Supporting Information 1 Figure S1: Analysis of top 10 GO functions of DEGs in D2, D7 and D14 samples; Figure S2: KEGG analysis of DEGs in D2, D7 and D14 samples; Figure S3: Analysis of key genes in the differentiation of iPSCs into cardiomyocytes. Figure S4: Schematic diagram of human BMP5 knockout model using CRISPR/Cas‐mediated lentiviral vector for genome engineering. Figure S5: Western blot to detect the BMP5 expression in BMP5 knockout hESCs group and control group. Figure S6: GSEA was conducted using GO pathways’ biological process branch as the gene sets of interest.; Figure S7: GSEA was conducted using GO pathways’ cellular component branch as the gene sets of interest; Figure S8: GSEA was conducted using GO pathways’ molecular function branch as the gene sets of interest. Table S1: The number of genes contained in different modules. Table S2. Important pathway enrichment analysis; Table S3: Transcriptome sequencing analysis of DEGs in BPM5‐KO. [file SCI-2026-5540587-s002.zip › Suporting information/datasets- WB(Review∩╝ë.pdf]
